# Multi-layer stratified oncology platform utilizing transcriptomics, prostate cancer organoids, and modeling of drug response

**DOI:** 10.1186/s13046-025-03540-2

**Published:** 2025-10-16

**Authors:** Juening Kang, Panagiotis Chouvardas, Andrew Maalouf, Daniel Hanhart, Laura Fernández Cerro, Wanli Cheng, Eva Compérat, Katja Ovchinnikova, Rahel Etter, Michaela Medová, Ulrich Schneeberger, Beat Roth, George N. Thalmann, Sofia Karkampouna, Marianna Kruithof-de Julio

**Affiliations:** 1 Urology Research Laboratory, Department for BioMedical Research, University of Bern, Bern, 3008 Switzerland; 2 Department of Urology, Inselspital, Bern University Hospital, University of Bern, Bern, 3010 Switzerland; 3 Department of Pathology, Medical University Hospital of Vienna, Vienna, Austria; 4 Department of Radiation Oncology, Inselspital Bern University Hospital, Bern, Switzerland; 5 Department for BioMedical Research, Radiation Oncology, University of Bern, Bern, Switzerland; 6 ZIO AG – Centre for Integrative Oncology, Hardturmstrasse 133, Zürich, CH-8005 Switzerland; 7 Translational Organoid Resource, Department for BioMedical Research, University of Bern, Bern, 3008 Switzerland

## Abstract

The high intra-patient heterogeneity in multifocal primary prostate cancer (PCa) has curtailed the efficacy of current treatment options. By employing twin biopsies from multiple lesions with matched patient-derived organoids (PDO) models, the PCa molecular heterogeneity was investigated. We utilized genomics, transcriptomics and machine learning (ML) approaches to elucidate and predict the underlying mechanisms of pharmacological heterogeneity. Our data indicate a vulnerability of primary PCa organoids for small molecule inhibitors targeting receptor tyrosine kinases (MET, ALK, SRC). By exploring gene expression data from matched parental tissue in an unsupervised manner, we identified two distinct clusters of samples. Interestingly, the PDO drug responses were significantly different between the two clusters for 4/11 compounds tested. We developed a transcriptomics-based, cluster prediction model, which can accurately stratify samples into the two clusters. Notably, our prediction model is based on tissue profiles, therefore, it can be utilized to rapidly evaluate new cases and suggest promising drug candidates, even when PDO derivation is not feasible. Taken together, we propose a novel flexible stratified oncology approach that can swiftly and accurately highlight promising drug vulnerabilities of PCa patients.

**Supplementary Information:**

The online version contains supplementary material available at 10.1186/s13046-025-03540-2.

## Introduction

Prostate cancer (PCa) is the second most common cancer in men worldwide, of a highly heterogeneous nature [1–3]. At the time of diagnosis, 60–90% of PCa patients are found to have multiple lesions, with different Gleason scores (GS), DNA ploidy, and genomic aberrations in key oncogenic /tumor suppressor genes, which can develop independently in the same prostate, posing a prognostic and therapeutic challenge [4, 5]. The current standard treatments for PCa are limited in terms of long-term effectiveness due to targeting the cancer as a bulk, without considering the multifocal nature of PCa and the intrinsic heterogeneity of each lesion [6]. Therefore, understanding the molecular (transcriptomic and genomic) heterogeneity of PCa and its impact on treatment response first at the cellular level and subsequently at the patient level is critical for optimizing personalized precision medicine decisions.

Recent studies have revealed that somatic mutations or DNA copy number changes are rarely shared among different tumor lesions within the same prostate [7]but also within a given lesion [8]. The presence of high inter- and intra-tumoral genomic heterogeneity and the sub-clonal diversity influences gene expression among distinct foci [9, 10]. Current tools fail to identify which properties of tumor foci are associated with higher risk for metastatic progression and /or lethal PCa. Such molecular complexity may affect treatment efficacy to androgen deprivation therapy (ADT) [11]. In fact, it has been shown that greater genomic diversity at the primary stage [12] is associated with resistance to ADT in patients. Molecular evolution analyses of independent tumor foci showed differential drug sensitivity to neoadjuvant ADT among the different foci in case report studies [13]. Inclusion of larger cohorts to dissect the responses of specific lesions pre- and post-treatment would be highly informative.

Such exploration of direct drug-mediated biologic responses in PCa, has been hampered by the slow progression of the disease and lack of suitable cell models, depicted by the limited number of PCa cell lines available, especially for primary stage, Androgen Receptor (AR) positive and treatment-naive PCa [14, 15]. This limitation can be circumvented using patient-derived organoids (PDOs), which have the potential to recapitulate cell type composition and tumor-specific molecular features. Yet they require fresh tissue, which is incompatible with routine histopathologic analysis that is prioritized for diagnostics. Previously, we, among others, have shown that PDOs from primary PCa may serve as a cellular model similar to primary cultures, which although not indefinitely proliferative, show higher establishment efficiency compared to i.e. 2D cell lines, and thus suitability for preclinical short-term patient-oriented studies [16–19].

High number of patients present with bone metastases at initial diagnosis, indicative of early metastatic dissemination parallel to primary PCa development [20]. Thus, identification of primary PCa traits, such as molecular features and therapeutic vulnerabilities is crucial for patient stratification, defining risk of metastatic aggressiveness and adjusting therapeutic management (more frequent surveillance, radiotherapy). In this study, a precision medicine approach of investigating functional drug responses of individual foci, in parallel with genomic and gene expression profiling of twin biopsies, has been employed. Using our previously developed methodology for PDO derivation from primary and advanced PCa, we assessed the in vitro chemosensitivity to standard-of-care (enzalutamide) and to a selected drug candidate panel of non-ADT related compounds that showed efficacy in PCa [17]. Multifocal features of PCa, including histological and molecular heterogeneity, as well as non-malignant epithelial areas were analysed to obtain information on subtle molecular, possibly premalignant features. We stratified the samples into distinct clusters based on their parental tissue transcriptomic profiles, associated them with drug response, and developed machine learning (ML) models to allow the classification of new samples. Taken together, we propose a new stratified oncology platform for the prediction of PCa drug vulnerabilities.

## Methods

### Patient specimen selection and preparation

The intact prostate was collected, cut transversally, and divided into four quadrants (top right “A”, bottom right “B”, bottom left “C and top left “D”) after patients underwent radical prostatectomy at the Inselspital, University Hospital in Bern. One core was taken from each lesion and each core was divided into two equal “mirror” pieces. One piece was taken for pathological assessment and the other piece was cryopreserved for further organoid generation. A cohort of 24 patient cases with a minimum of one core with Gleason score (GS) ≥ 6 were selected for the study, according to the histopathological assessment by a board-certified pathologist. All patients included in this study provided written informed consent under the ethical approval by Swissethics and Cantonal Ethical Committee, Bern, Switzerland (EK BE 17/02295).

### Tissue dissociation and organoid culture

Cryopreserved tissue was thawed in Basis medium (Advanced DMEM F12 Serum Free medium (Thermo Fisher Scientific, 12634010) containing 10 mM Hepes (Thermo Fisher Scientific, 15630080), 2 mM GlutaMAX supplement (Thermo Fisher Scientific, 35050061), and 100 µg/ml Primocin (InVivoGen, ant-pm-1)). After mechanical disruption the tissue was washed in Basis medium (300 rcf, 5 min) and incubated in enzyme mix for tissue dissociation (collagenase type II enzyme mix (Gibco, 17101015), 220 U/ml dissolved in Basis medium, 15 µg/ml DNase (Roche, 10104159001), 10 µM Y-27632-HCl Rock inhibitor (Selleckchem, S1049), 200 U/ml collagenase type IV (Gibco, 17104019), 0.6 U/ml Dispase II (Sigma, D4693-1G), 100 U/ml Hyaluronidase (Sigma, H3506-500MG), 3mM Calcium chloride dihydrate (Sigma, C7902-500G)). Enzyme mix volume was adjusted so that the tissue volume does not exceed 1/10 of the total volume and tissue was incubated at 37 °C for 1.5–2.5 h with pipette mixing every 30 min. After digestion of large pieces was completed, the suspension was spun down (300 rcf, 5 min) and washed twice with 5 ml Basis medium (300 rcf, 5 min). The pellet was suspended and further digested with 2 ml TrypLE™ Express Enzyme (Thermo Fisher Scientific, 12605028), incubated at 37 °C for 20–30 min. The cell suspension was counted to determine seeding density and then washed twice with 5 ml Basis medium (300 rcf, 5 min). Cell pellet was reconstituted in 2-2.5 ml PCa organoid medium and seeded in ultra-low attachment (ULA) plates, with 300,000–500,000 cells per well of 6-well plate (Corning, 3471). PCa organoid culture medium contains the following reagents: Basis medium with 10 µM Y-27,632-HCl (Selleckchem, S1049), 5% fetal calf serum (Gibco, 10270-106), 1× B-27 supplement (Thermo Fisher Scientific, 17504044), 10 mM Nicotinamide (Sigma, N0636-100G), 500 ng/ml Rspondin (Peprotech, 120 − 38), 1.25 mM N-acetyl-cysteine (Sigma, A9165), 10 µM SB202190 (Selleckchem, S1077), 100 ng/ml Noggin (Peprotech, 250 − 38), 500 nM A83-01 (Tocris, 2939), 10 nM DHT (Fluka Chemica, 10300), 10 ng/ml Wnt3a (Peprotech, 315 − 20), 50 ng/ml HGF (Peprotech, 100 − 39), 50 ng/ml EGF (Peprotech, AF-100-15), 10 ng/ml FGF10 (Peprotech, 100 − 26), 1 ng/ml FGF2 (Peprotech, 100-18B), 1 µM PGE2 (Tocris, 2296). Medium was prepared and kept at 4 °C for no longer than 7 days.

### Organoid quantification

Organoid images were acquired using an inverted brightfield microscope (Olympus, U-TV0.5XC-3) and analysed using Image Analysis software ImageJ (v1.53t). Organoids were manually counted and classified in three categories as solid, hollow, or mixed depending on the respective morphological features [21, 22]. Furthermore, each organoid was manually selected as a region of interest, and the total area and dimensions were measured. The diameter of each organoid was obtained by calculating the average of the width and height.

### Immunofluorescence staining

FFPE sections (5 μm) were deparaffinised and used for heat mediated antigen retrieval (citrate buffer pH 6, Vector labs). Sections were blocked for 60 min (min), RT in 1%BSA in PBS − 0.1% Tween20 before incubation with primary antibodies (diluted in 1% BSA in PBS − 0.1% Tween20) at 4 °C overnight. Sections were washed once in PBS − 0.1% Tween20 for 5 min, then twice in PBS for 5 min before incubation at RT for 90 min with secondary antibody, diluted in PBS. Sections were then washed twice in PBS for 5 min, then incubated in a solution of TO-PRO-3 in PBS for 10 min. Sections were then washed twice in PBS for 5 min and mounted using Prolong Gold anti-fade mounting medium (Thermofisher Scientific). The following antibodies were used:

**Table:** 

Dilution	Antibody	Company	Catalog No	Clone
1 to 100	E-Cadherin	R&D Systems	AF648	-
1 to 400	Ki67	Gene Tex	GTX16667	SP6
1 to 100	P63	Santa Cruz	sc-25,268	D-9
1 to 100	CK8	abcam	ab53280	EP1628Y
1 to 100	CK5/6	Chemicon Milipore	MAB1620	D516B4
1 to 100	AR	abcam	ab133273	EPR1535(2)
1 to 500	TO-PRO-3	Thermo Fisher	T3605	-

### Immunohistochemistry and image analysis

Formalin-fixed paraffin-embedded sections were deparaffinized and rehydrated. The heat-induced antigen retrieval was performed in citrate buffer for 30 min at 95 °C. Afterwards, the sections were incubated for 10 min in peroxidase blocking solution 3% hydrogen peroxide (Merck Millipore, 107209), and for 1 h in 1% normal goat serum (DAKO, X0907). The primary antibody targeting MET Tyr1234/5 (1:150, clone D26, CST, 3077; RRID: AB_2143884) was added overnight at + 4 °C. The next day, the visualization was achieved through the consecutive incubation of the secondary antibody goat biotinylated anti-rabbit IgG (Vector Laboratories, PK-6101) for 1 h, the ABC reagent solution (Vector Laboratories, PK-6101) for 30 min, and the 3,3’-diaminobenzidine (DAB) solution (Vector Laboratories, SK-4100) for 10 min. Hematoxylin was used for counterstaining and the Eukitt mounting medium (Sigma-Aldrich, 03989) to cover the samples. The slides were scanned with a Pannoramic 250 Flash III Scanner (3DHISTECH).

Image analysis and quantification of phosphorylated MET levels was performed using QuPath source software [23]. Whole slide areas were imported into Qupath (version-0-6-0) and an ROI selection including a positive area, negative area and background was used to estimate the most accurate stain vectors (automatic threshold was used). Cell detection was done based on Optical Density Sum. Positive cell classification was obtained using the Cell DAB detection option using a minimum threshold of 0.2. The positive cell detection (% positive cells over the total) was performed using a whole section annotation, excluding only the section edges, and with the same threshold for all samples.

### Drug response assay

Organoids were dissociated with TrypLE™ Express Enzyme (Thermo Fisher Scientific, 12605028) once they formed and seeded in 348 well ULA plates with 20 µl PCa organoid medium. After 48 h of culture, 20 µl of drug compounds/ vehicle containing 2x of the final concentration in PCa organoid medium, were added to organoid cultures. After 48 h of drug treatment, CellTiter-Glo 3D assay (Promega, G9682) was used to measure cell viability. The CellTiter-Glo 3D reagent was added (40 µl per well) to the assay plates. Plates were subsequently orbitally rotated (5 min at 300 rpm, plate reader) to allow mechanical disruption of the organoids and incubated at 37 °C for 25 min for complete cell lysis. After incubation, luminescence was measured using Varioskan Lux Microplate Reader. Raw counts were used to generate fold- changes with respect to the average of vehicle (DMSO) raw values. The following compounds and concentrations were used:

**Table:** 

Compound	Company	Catalog No	Final Concentration
DMSO	Sigma Aldrich	D4540	0.1%
Bosutinib	Selleckchem	S1014	10 µM
Crizotinib	Selleckchem	S1068	10 µM
Daunorubicin	Selleckchem	S3035	10 µM
Doxorubicin	Selleckchem	S1208	10 µM
Enzalutamide	Selleckchem	S1250	6 µM
Epirubicin	Selleckchem	S1223	1 µM
Erlotinib	Selleckchem	S7786	10 µM
Ponatinib	Selleckchem	S1490	10 µM
Rapamycin	Selleckchem	S1039	10 µM
Sunitinib	Selleckchem	S7781	10 µM
Temsirolimus	Selleckchem	S1044	10 µM

The drug screen on patient-derived xenograft (PDX) - derived organoids (PDXOs) (PNPCa, BM18 and PNPCa) was performed as previously described [17]. For IC50 estimation, organoid viability measurements were normalized to the DMSO vehicle and data were log transformed and used for a nonlinear fit (log(inhibitor) vs. normalized response-variable slope).

### Ion torrent sequencing

DNA from blood and organoids was extracted using the DNeasy Blood and tissue kit (Qiagen, 69504). DNA from FFPE material was extracted with Maxwell^®^ 16 FFPE plus LEV DNA Purification Kit (Promega, AS1135). Targeted sequencing of PDOs and their corresponding parental tissues was performed using a custom panel targeting the most frequently mutated genes in prostate cancers [9].

### Genomic analysis

Somatic variant calling was performed using the *PipeIT2* (version 2.0.0) workflow [25]. Briefly, it includes somatic variant calling using the *Torrent Variant Caller (TVC)*, normalizes multiallelic variants, and filters variants in a multistep process. In both tumor-germline and tumor-only analyses, a bed file was provided to restrict variant calling to targeted regions, and non-coding variants were included in the final output. Following the variant calling, all mutations were annotated using *snpEff* (version 5.0) [26]and key fields were extracted using SnpSift [27]. The resulting annotated variant call format (VCF) files were converted into Mutation Annotation Format (MAF) files using *vcf2maf* (version 2.4) [28].

### RNA sequencing

For RNA sequencing of FPPE tissues we used an exome capture- based approach to account for the RNA degradation issues [24]. RNA from regions of interest was extracted from FFPE material using the Ambion Kit. The NEBNext Ultra II Directional RNA Library Prep Kit for Illumina (NEB #E7760S/L) was employed for fragmentation and cDNA synthesis. Depending on RNA quality, two fragmentation conditions were used: 10 min at 94 °C for samples with RQN ≥ 4, and 5 min priming at 65 °C for others. Following fragmentation, cDNA synthesis took place. Quality control was subsequently conducted using Qubit to ascertain the starting concentration.

Sample preparation and hybridization capture were executed in line with the manufacturer’s protocol for the SureSelectXT Low Input Target Enrichment System for Illumina Paired-End Sequencing Library (protocol v1.8, G9703-90000). Briefly, adapters were ligated to the fragmented cDNA, followed by PCR amplification. Prior to the PCR amplification a USER-enzyme step was executed (1 µl, 15 min at 37 °C). The cDNA was then purified using 1x Ampure Beads.

For the hybridization process, Agilent SureSelectXT Human All Exon v7 Capture Library (5191 − 4006) baits were utilized. This step was conducted overnight. After the hybridization, a capture wash was carried out, following by a concluding PCR amplification. Final quality and yield assessments were performed using both Qubit and the Fragment Analyzer. Clustering and DNA sequencing using the NovaSeq6000 was performed according to manufacturer’s protocols.

### Transcriptomics analysis and clustering

RNA sequencing data were analyzed using the Nextflow (version 23.04.1) *rnaseq nf-core* workflow (version 3.10) [29, 30]. Quality control was performed with *FastQc *(version 0.11.9) [31] and *RSEQC* (version 3.0.1) [32]. The sequencing reads were aligned against hg38 human reference genome using *STAR* (version 2.7.9a) [33] and the gene expression was quantified using *Salmon* (version 1.9.0) [34] and the 108 version of Ensembl gene annotation. Downstream analyses were performed in *R* (version 4.4.2) utilizing *DESeq2* (version 1.46.0) [35] normalized counts. Principal components analysis (PCA) was followed by estimation of the % of variance explained by the individual principal components (PCs). Applying a threshold of 5% variance explained, 3 PCs were kept for the clustering analysis. Silhouette method from the *factoextrA *[36] package (version 1.0.7) identified 2 as the optimal number of clusters to partition the patients’ gene expression profiles. Clustering was performed with the kmeans algorithm and the 2 clusters were named C1 and C2, respectively. *PROGENy* pathway activity [37] was estimated using the *viper* algorithm via the wrapper functions of the *decoupleR* (version 2.9.7) package [38]. Master Regulator analysis was done with the same package and using the *CollecTRI* set of regulons [39].

### Machine learning

Machine learning modeling was performed with the *tidymodels* package (version 1.2.0) [40] with the task of classifying the samples in the two clusters. The pathways’ activity was used as features. The data was split in training and test sets (75% and 25% respectively). The optimal algorithm and modeling were selected by cross validation and by testing different algorithms (i.e. multinomial regression, support vector machines, random forest, nearest neighbor and xgboost). Multinomial regression performed the best and was therefore selected for the final training. The remaining 25% of the samples were used for an unbiased estimation of the model’s accuracy.

### Cluster signatures and TCGA data analysis

We performed differential expression among the two clusters using *DESeq2.* The differentially expressed genes (DEGs) were identified with an adjusted p-value cutoff of 0.05 and an absolute log2 fold change threshold of 1. We focused on the protein-coding genes using *biomart* [41]. The top 50 DEGs per cluster were then explored in TCGA data using *Gene Set Cancer Analysis (GSCA)* webserver [42]. Pathway and signature activity was estimated with *Gene Set Variation Analysis (GSVA)* [43]. Significant associations to pathways were selected with a false discovery rate (FDR) cutoff of 0.05. Survival analysis was performed in *R*, and the significance was estimated with a log-rank test.

## Results

### Generation and culture of PDOs from heterogenous primary PCa clinical samples

**Fig. 1:**
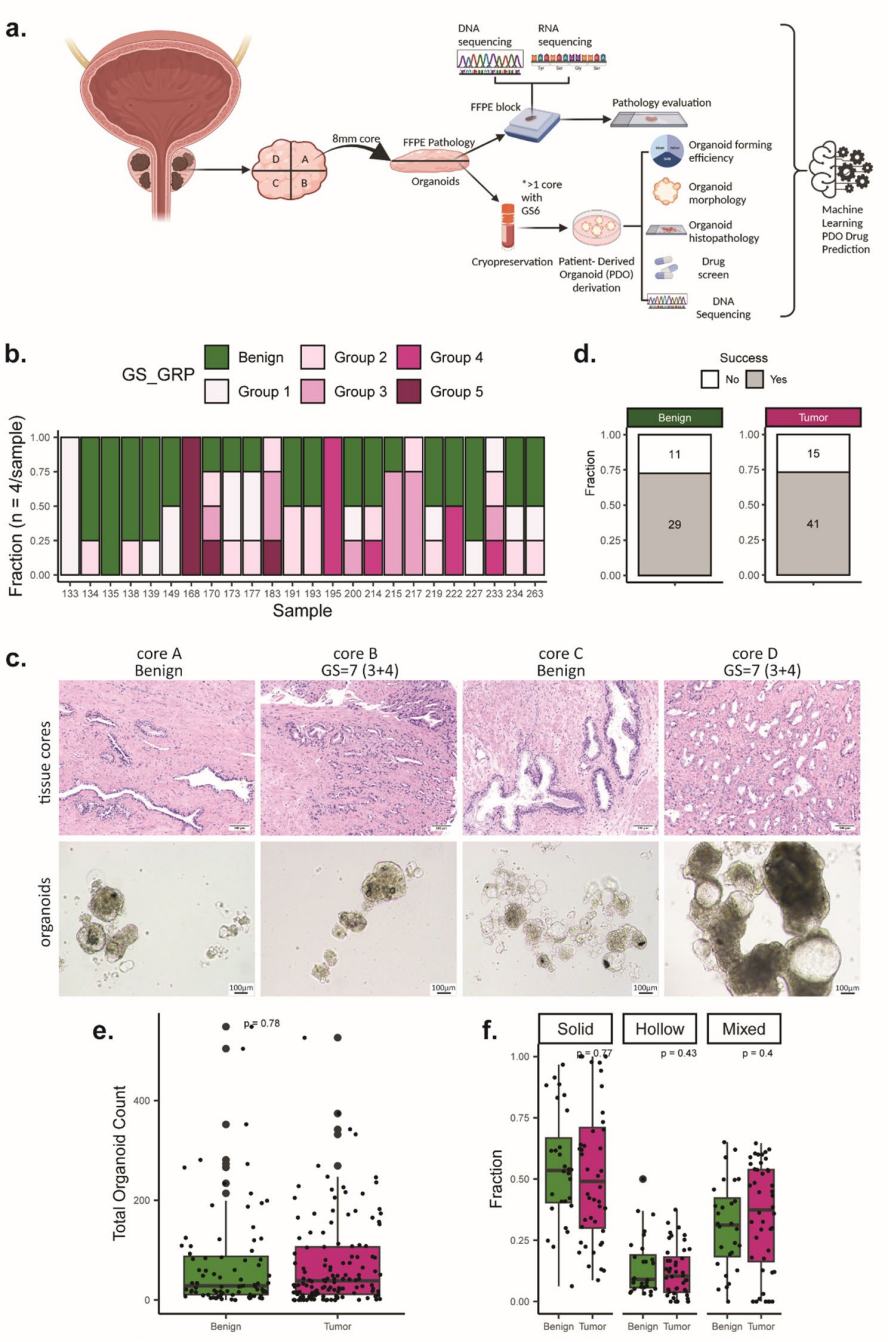
Derivation and culture of patient-derived organoids (PDOs) from multifocal primary prostate cancer (PCa). (**a**) Scheme of the experimental protocol for multifocal primary PCa organoid derivation and downstream analysis. Created in https://BioRender.com. (**b**) Gleason grade group reported for each of the tissue cores. (**c**) Morphology of multifocal primary PCa and matched PDOs (passage (p) 0) from a representative case. H&E images of primary tumor and brightfield images of deriving PDOs. (**d**) Fraction of successful and PDO formation in relation to the tissue of origin (benign or tumor category). Total number of core samples: histopathologically defined benign (*N* = 40, upper plot) and tumor cores (*N* = 56, bottom plot) were used. (**e**) Quantification of yield of total organoid numbers obtained from benign vs. tumor cores (Wilcoxon test, *p* = 0.78). (**f**) Fractions of distinct PDO morphologies from benign versus tumor cores (average number of solid morphology in benign vs. tumor cores: Wilcoxon test *p* = 0.77; average number of hollow morphology in benign vs. tumor cores: Wilcoxon test *p* = 0.43; average number of solid morphology in benign vs. tumor cores: Wilcoxon test *p* = 0.4)

Tissues from radical prostatectomies were divided in quadrants and four core biopsies were obtained from each quadrant. The cores were obtained as twin/mirror biopsies; one half of the core was processed for histology and omics, and the other half was processed for cell derivation, PDO establishment, genomic characterization and drug screening (Fig. 1a). The cohort consists of 24 patient cases, with 2–4 cores per case, of which at least one has tumor content as assessed by expert pathologist (Sup. Table 1). The distribution of GS in this patient cohort is grade group 1 (4.16%), group 2 (50%), group 3 (33.3%), group 4 (8.3%), group 5 (4.16%) (Sup. Figure 1a). The distribution of GS of the core lesions in this patient cohort is grade group 1 (17%), group 2 (16%), group 3 (11%), group 4 (8%), group 5 (6%) with the remaining cores being benign (Fig. 1b). Varying GS and tumor content emerged when assessing the distinct foci from each core (Sup. Figure 1b). 19/24 patients have at least 2 tumor lesions. 12 out of those 19 have at least 2 tumor lesions with different GS, highlighting the high intra-patient heterogeneity.

PDOs were generated from four distinct areas (designated as cores A-D) of each prostate (Fig. 1a-b) using our previously established organoid methodology in low adherence conditions (ultra low attachment cultures, absence of extracellular matrix), shown to effectively retain tumor luminal cells from human primary and metastatic clinical specimens, as well as from patient-derived xenograft models [17]. One modification from the original methodology was that we included additional proteases to the collagenase type II, to degrade the dense prostate tissue more effectively. This was a necessary step for tissue cores of primary PCa, compared to needle biopsies, due to the high content of matrix proteins and tissue stiffness, and it allowed to keep the tissue digestion short and retrieve single cells with high viability. Histopathology of PDOs was compared to histopathological features of the matching tissue cores for the distinct tumor foci and benign areas (Fig. 1c).

The PDO forming efficiency was equally successful between non-tumor-containing cores (*N* = 29/40, 72.5%) and tumor-containing cores (*N* = 41/56, 73.2%, two-sided Fisher’s test, *p*-value > 0.99), (Fig. 1d). PCa PDOs formed within 5–11 days (Sup. Figure 1c), while failure to generate PDOs did not correlate with the initial yield of viable cells (Sup. Figure 1d, unpaired t test, *p*-value = 0.2658). Similarly, tumor-containing cores had a similar yield of organoid number as the benign ones (Fig. 1e, p *=* 0.78).

PDOs exhibited three distinct morphological patterns: solid, hollow/luminal, and mixed. We then investigated whether an association of morphology with tumor features exists, as similarly shown for murine PCa organoids with solid structures typical of adenocarcinoma and luminal structures composed mainly of normal cells [22]. Solid organoids were densely packed epithelial structures without a lumen, hollow organoids consisted of a lumen surrounded by a single or multiple layers of cells, while mixed structures presented both solid and hollow features (Fig. 1c, core D). In this PDO cohort, solid structures were predominantly present from all distinct foci, irrespective of parental tissue histopathological features (tumor/benign) (Fig. 1f, p *=* 0.77). No statistically significant differences were found in the fraction of either hollow or mixed PDOs in relation to tissue origin (Fig. 1f, p *=* 0.43 and *p =* 0.4, respectively). The average number (Sup. Figure 1e) and diameter (Sup. Figure 1f) of each PDO morphology, that presented from either tumor or benign cores, was overall similar among different GS (Sup. Figure 1e-f, left) and TC groups (Sup. Figure 1e-f, right), indicating no direct correlation of PDO morphology with presence of carcinoma.

Histopathology of PDOs in FFPE sections was reviewed by a board-certified pathologist. Overall, the PDOs had well defined structures with clear limits (Sup. Figure 2a), with several tumor features with atypia, high nucleus-plasma ratio and nuclear hyperchromasia (Sup. Figure 2b). Tumor content of the organoid structures was 40%-80% and found at similar levels among PDOs from benign (*n* = 6), GS6 (*n* = 4) or GS7 (*n* = 4) tumors (Sup. Figure 2c), indicative of potential tumor cell selection in PDO cultures.

### PDOs retained the histological and genomic traits of parental tissues

**Fig. 2:**
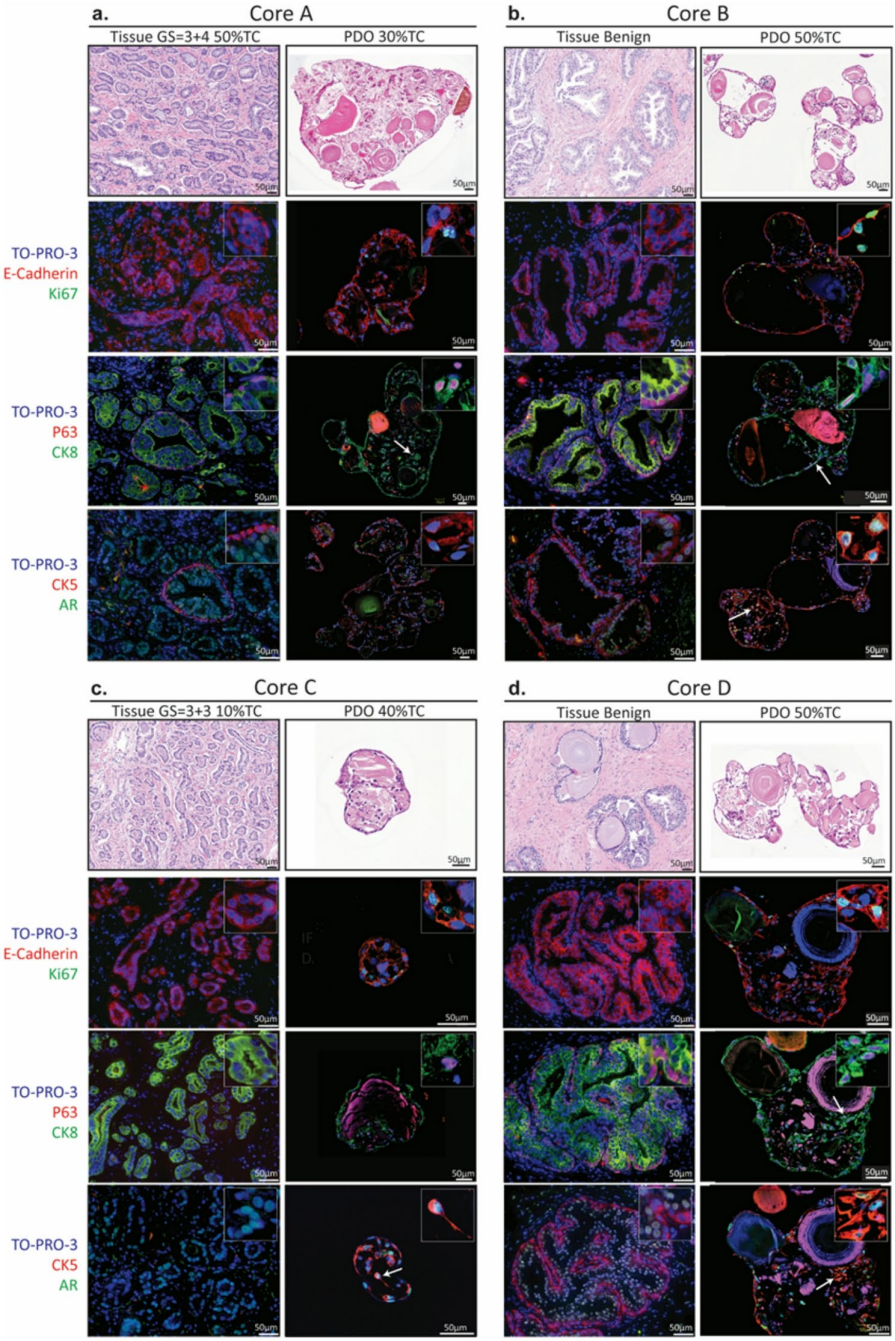
Prostate cancer (PCa) patient-derived organoids (PDOs) originating from independent tumor/benign foci recapitulate histopathological features in vitro Representative core tissues and matching PDOs from the same case; of histopathologically defined tumor (**a** and **c**, PCa263 core A and core C) and benign (**b** and **d**, PCa263 core B and core D). (**a-d**) H&E and immunofluorescence stainings on FFPE tissue and PDO sections. Top panel: proliferation marker Ki67 (green), epithelial marker E-cadherin (red). Middle panel: luminal marker cytokeratin-8, CK8 (green), basal marker p63 (red). Bottom panel: luminal marker androgen receptor AR (green), basal marker cytokeratin-5, CK5 (red). TOPRO-3 (blue) marks the nuclei. White arrows indicate examples of cells expressing both basal and luminal markers. Scale bar: 50 μm

To compare the histological features of PDOs with their parental tissue, as well as between tumor and non-tumor containing cores, the expression of prostate epithelial lineage markers in both PDOs and their parental tissue was assessed through immunofluorescence (IF) staining. We investigated an array of markers, encompassing epithelial (E-Cadherin), luminal (CK8 and AR), basal (p63 and CK5), and the proliferation marker (Ki67) in two representative cases (Fig. 2, Sup. Figure 3).

The expression of E-Cadherin marker vouched for the epithelial origin of the prostate gland in both the parental tissue and PDOs. The presence of Ki67-positive cells in PDOs indicated their proliferative capacity; however, their absence in parental tissue might stem from the low proliferative index of primary PCa, compatible with the slow disease progression.

The expression of both luminal (CK8 and AR) and basal (P63 and CK5) markers substantiated the co-existence of the two prostate epithelial lineages in the organoid cultures. Furthermore, luminal CK8 and AR positive cells were identified around a lumen-like gland in PDOs, surrounded by basal P63 and CK5 positive cells. This was consistent across benign tissues (Fig. 2b, d, Sup. Figure 3b), as well as PDOs from both tumor and benign tissues, although tumor tissues exhibited predominant luminal (CK8 and AR positive) cells with only a few surrounding basal (P63 and CK5 positive) cells (Fig. 2a, c, Sup. Figure 3a). The predominantly luminal tumor cell phenotype of the primary PCa PDOs in this cohort is in line with our previous findings in primary and metastatic PDOs and is due to the culture methodology we previously established under low adherence conditions and absence of extracellular matrix (e.g. Matrigel). Intriguingly, an intermediate cell population, referred to as intermediate or transit amplifying cells [44, 45] that co-expresses luminal and basal markers (CK8 and P63, AR and CK5) was also identified in organoids (Fig. 2, pointed with white arrows), possibly indicating an intermediate population.

**Fig. 3:**
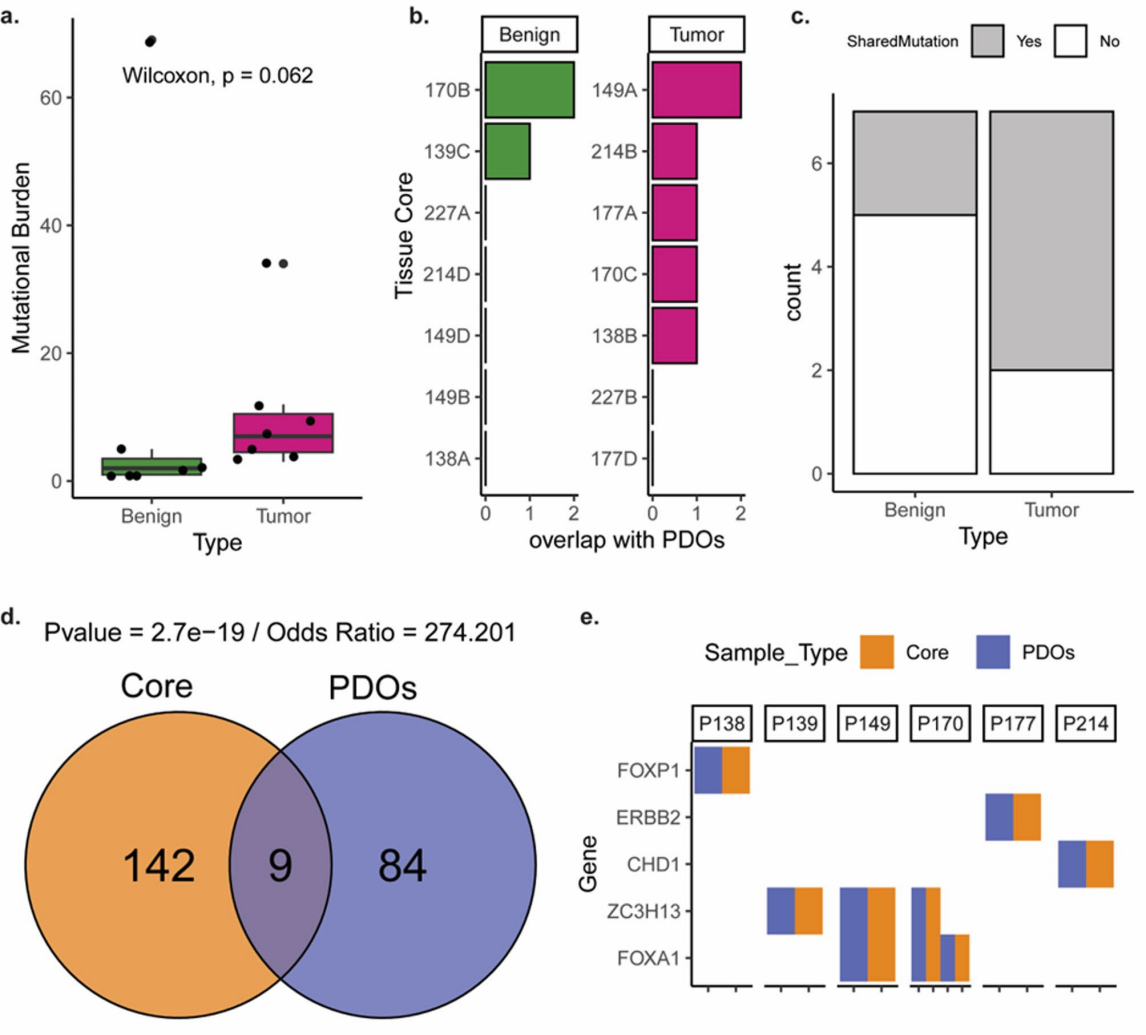
Mutational characterization of multifocal PCa tissue cores and organoids with matched blood control (**a**) Distribution of mutational burden on tumor and benign cores as defined by pathological evaluation (Wilcoxon test, *p =* 0.062). (**b**) Overlap of somatic mutations among matched cores and organoids of different patients. (**c**) Summary of overlap of somatic mutations between matched core and organoid, for benign and tumor cores. (**d**) Venn diagram representation of overlapping and non-overlapping gene mutations in all tissue cores versus all organoid samples and statistical significance of overlap (Fisher’s exact test, *p =* 2.7e-19). (**e**) Genes that harbour shared mutations in different cases (P138, P139, P149, P170, P177, P214) in tissues and organoids

A subset of PDOs and their matched tissue cores (*N* = 14) were subjected to targeted genomic sequencing using a PCa panel and matched germline blood controls [9]. The overall mutational burden is relatively low (mean burden ~ 11 mutations per sample), as expected for the primary stage of the disease, with a slightly higher trend of mutational burden in tumor cores than in benign, albeit non-significant (Fig. 3a, Wilcoxon`s test, *p =* 0.062). PCa somatic mutations were identified in the majority of the tumor cores (*N* = 5/7), and in some of the histopathologically-defined benign cores (*N* = 2/7) (Fig. 3b-c). 9 mutations were shared between the parental tissue and corresponding PDOs (Fig. 3d-e, Fisher’s exact test, *p =* 2.7e-19)., in frequently mutated, PCa-associated genes such as FOXP1, ERBB2, CHD1, ZC3H13 and FOXA1 (Fig. 3e). In a separate cohort (*N* = 13) of tissues and PDOs without matched germline controls, a higher mutational burden (Sup. Figure 4a), accompanied by higher overlap of mutations between cores and PDOs was observed (Sup. Figure 4b), irrespective of the histopathology of the cores. This observation highlights the need for germline controls to avoid the overestimation of somatic mutations and subsequent conclusions derived by such data.

Although the cohort revealed a limited number of mutations, there is pronounced genomic heterogeneity in benign regions in multifocal primary PCa. Interestingly, a few cores pathologically classified as benign still exhibited genomic mutations (*N* = 2/7), which were partly shared with their deriving PDOs (Fig. 3a-b, Sup. Figure 4a-b). This is in line with a higher number of copy number variations reported in hundreds of genes in several histopathologically-defined benign areas, adjacent to tumor foci, albeit not as high as in tumor areas [46]. These observations indicate the genomic heterogeneity and aberrations that occur under the surface in benign, tumor-adjacent tissues, which indicate oncogenic transformations prior to morphological changes [47]. Genomic profiling combined with pathological evaluation thus offers a more comprehensive characterization of benign and tumor lesions in multifocal PCa by complementing the pathological assessment.

### Transcriptomic analysis offers a comprehensive depiction of PCa heterogeneity and molecular associations with PDO drug response

We investigated the presence of transcriptomic diversity of multifocal primary PCa, by subjecting the parental tissues of tumor foci and benign areas to RNA-sequencing (RNA-Seq). To improve the output of RNA-Seq impacted by the RNA degradation in the FFPE tissues, we compared two library preparations on the same samples, either ribosomal RNA depletion (Sup. Figure 5a, c) or random primer cDNA synthesis in combination with targeted sequencing by hybridization with 5` prime human-specific probes (Sup. Figure 5b, d). Higher number of mapped reads (Sup. Figure 5b) and exonic regions (Sup. Figure 5d) was achieved with the latter method, compared to ribosomal RNA depletion and total RNA-Seq (Sup. Figure 5a and 5c, respectively), which was eventually applied to all subsequent FFPE tissue core samples for gene expression analysis.

**Fig. 4:**
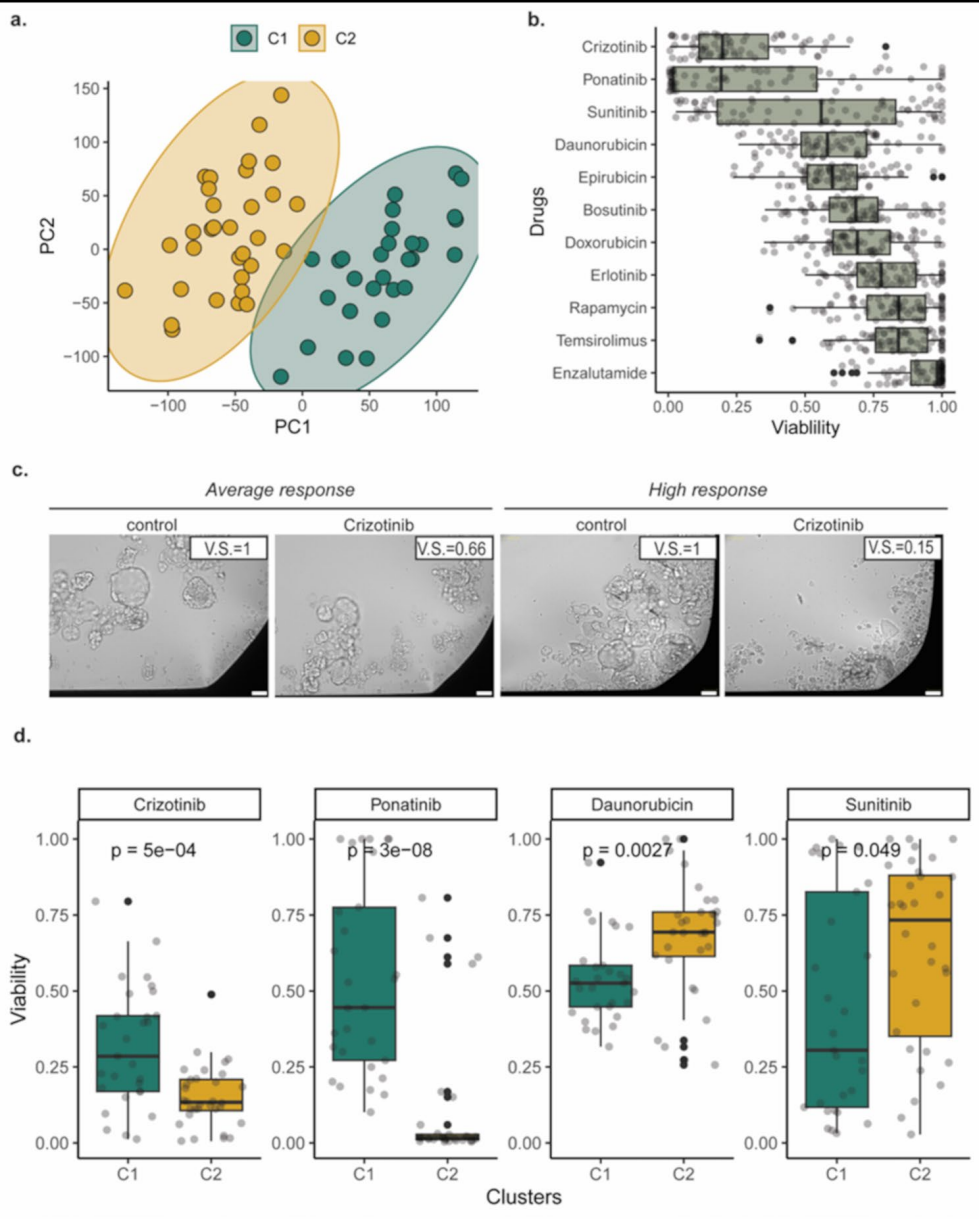
Transcriptomic profiles on parental tissues can discriminate two clusters of drug responses in matched PDOs, highlighting drug vulnerabilities to receptor tyrosine kinase inhibitors (**a**) Principal component analysis (PCA) for gene expression in FFPE parental tissues followed by unsupervised clustering (k-means). (**b**) PCa drug panel testing to assess drug efficacy on PDO viability. The ATP-mediated cell viability values were distributed across our cohort (*n* = 63 PDOs from distinct tumor/benign cores derived from 24 different patient samples) and the drug panel tested. Viability from 0 to 1, over the vehicle control values of each sample. (**c**) Brightfield Images of PDO morphology after 48 h drug treatment. *Left panel*: a representative case of average drug response to crizotinib (viability score (V.C) 0.66 versus the control (V.C.=1). *Right panel*: a representative case of high response to crizotinib (V.C.=0.15). Control condition is treatment with a DMSO vehicle. (**d**) For the four most effective drug compounds (PDO viability median ~ 0.50 and lower), the identified transcriptomic signatures, clusters C1 and C2, discriminate two groups of drug response (Wilcoxon test C1 vs. C2: crizotinib *p =* 5e-04, ponatinib *p =* 3e-08, daunorubicin *p =* 0.0027, sunitinib *p =* 0.049)

Principal component analysis (PCA) showed marked distinctions in gene expression profiles across different patients (Fig. 4a, Sup. Figure 6a). Differential expression analysis among tumor and benign cores revealed 951 differentially expressed genes (Sup. Figure 6b). Differential expression analysis was followed by Master Regulator (MR) analysis and we identified MRs, known to be related to PCa, overactivated in the tumor cores (Sup. Figure 6c), such as AR, validating the accuracy of our profiling.

We next, sought to explore if the samples can classified into distinct strata, in a data-driven manner. We utilized the first 3 principal components which individually explain more than 5% of the variance (Sup. Figure 6d) and the Silhouette method and identified that 2 is the optimal number of clusters (Sup. Figure 6e). The PCA-based approach allows the reduction of the dimensionality and exploration of the data. We subsequently employed the k-means clustering analysis and categorized the cores into two distinct clusters (Fig. 4a). We evaluated how stable the clustering is with a bootstrap-based approach and observed that the clusters are extremely stable (mean Jaccard similarity across bootstraps ~ 0.95). The cores from the same patients are predominantly grouped in the same cluster (Sup. Figure 6f), suggesting that our data-driven and unbiased approach overcomes the limitations of intra-patient heterogeneity.

Considering the intrinsic heterogeneous histological and molecular features observed in primary PCa, different tumor foci within the same prostate may possess distinct biological properties and molecular features, which may consequently result in diverse responses to therapeutic drugs. Therefore, we sought to synchronously determine the transcriptomic profile and with the functional variability in terms of drug response, utilizing mirror biopsies (Sup. Figure 7a-b). To address this, we conducted drug screening on PDOs using 11 selected compounds based on our previous study [17] including standard-of-care (SOC, Enzalutamide) as well as different FDA-approved drugs with indications for other solid cancers such as tyrosine kinase Inhibitors (TKIs, bosutinib, crizotinib, ponatinib, etc.), Anthracyclines (daunorubicin, doxorubicin, and epirubicin), and mTOR inhibitors (rapamycin, temsirolimus) (Sup. Table 2). These compounds previously showed efficacy in organoids from primary and advanced patient samples and patient-derived xenografts of PCa. The timing of organoid formation and duration of drug treatment was previously optimized, with a 48–72 h found to be optimum for most samples tested (Sup. Figure 7b-c). A range of concentrations including the Cmax, were tested in our previous study in PDXOs (Sup. Figure 7d), from which the minimum effective dose for androgen dependent tumor organoids, that led to statistically significant reduction in viability, was used for this present study. The effective dose for the PDXOs and PDOs was often higher than the Cmax, which was also validated in dose response curves (Sup. Figure 7d-e). In this present cohort, three TKIs (crizotinib, ponatinib, and sunitinib), predominantly targeting oncogenic receptor tyrosine kinases (RTKs), demonstrated broad effectiveness, representing the most effective drugs. crizotinib targets specifically the activation of c-MET, VEGFR, RET, AXL, ALK, ROS1, ponatinib targets SRC, VEGFR, FGFR, PDGF receptors and sunitinib targets RET, VEGFR, FLT3, and PDGF receptors [48]. Conversely, enzalutamide, along with mTOR inhibitors (rapamycin and temsirolimus) and EGFR inhibitor erlotinib, indicated an overall lower efficacy in reducing PDOs viability (Fig. 4b). The lower efficacy of enzalutamide is probably attributed to the high DHT dose in the organoid medium and the short time frame (48 h) to induce cell apoptosis/ inhibit proliferation. The drug treatment is performed on formed organoids, after seeding of equal number of single cells, to ensure homogeneity across replicates, which were cultured for 48 h to allow organoid formation (Sup. Figure 7b); representative images of the PDO morphology during the timing of the assay are shown in Sup. Figure 7c). Overall, crizotinib treatment was highly effective, with 50% ATP-mediated viability reduction for most tested PDOs (Fig. 4b). The viability readout is matching the morphologic changes observed in the PDOs after 48 h crizotinib treatment, with higher viability score (V.S) (Fig. 4c, V.S = 0.66 average response) showing intact PDO structures compared to lower V.S that shows increased dissociation of the organoid structure, increased number of single cells and debris (Fig. 4c, V.S = 0.15 high response). Expression of the crizotinib target, MET, in its activated state (tyrosine phorsphorylation pTyr1234) was confirmed in matched samples that were subjected to FFPE RNASeq (C1 vs. C2 clustering) (Sup. Figure 8a), pMET immunodetection (Sup. Figure 8a-c), along with organoid drug responses. Higher percentage of pMET positive cells in the tissue (Sup. Figure 8c) was inversely correlated with organoid viability (overall correlation (*r* = -0.66,* p value* = 0.071), individual cluster correlation C1 (*r* = -0.8642,* p value* = 0.136), and C2 (*r* = -0.3871,* p value* = 0.613) (Sup. Figure 8d) indicating that crizotinib efficacy is higher upon increased activation of its RTK target.

To rationalize the overall high efficacy of RTK inhibitors in the absence of genomic mutations, we specifically checked the gene expression profile or RTK ligands and receptors. RTK ligands related to hypoxia and angiogenesis are statistically significant, differentially expressed among the two clusters C1 and C2 (Sup. Figure 9a, upper). The high sensitivity of PCa PDOs to crizotinib (≥ 50% reduction of viability in most samples) is possibly due to presence of the target, both at protein and mRNA level. The RTK MET receptor mRNA expressed in both clusters. MET upregulation in C1 vs. C2 (Sup. Figure 9a, bottom, log2Fc = 0.5), is showing higher organoid viability (median viability C1 = 0.28, vs. C2 = 0.134). Instead, no significant difference was detected in the MET receptor ligand, HGF (Sup. Figure 9a, upper) or the other crizotinib targets ALK and ROS1 (Sup. Figure 9a, bottom). Our results suggest that targeting MET in primary PCa tumors with MET overactivation could be an effective treatment option. Other RTK receptors, such as Wnt receptors (RYK, ROR1), VEGF receptors (FLT1, KDR), and RET and its ligand BDNF, are upregulated in C1 when compared to C2 (Sup. Figure 9a). This possibly indicates higher protein accumulation and activation through tyrosine phosphorylation, thus making them sensitive to RET-and VEGF-R targeting sunitinib and to VEGF-R targeting ponatinib.

When combining the identified clusters and the drug response results, we observed that the clusters show differential response only for the 4 most potent drugs; crizotinib, ponatinib, daunorubicin and sunitinib (Fig. 4d, Sup. Figure 9b). The two most potent overall TKIs, crizotinib and ponatinib, follow the same trend, with the C2 cluster showing the highest response to both drugs (*p =* 5e-04 and *p =* 3e-08, respectively) (Fig. 4d, left). Conversely, C2 cluster is responding significantly less effectively to daunorubicin and sunitinib (Fig. 4d, right), with sunitinib showing more heterogeneous responses. Regarding other RTKs such as EGFR, no transcript levels were significantly altered (data not shown), and no major efficacy of TKI erlotinib on PDO viability (Fig. 4b) was observed. Thus, advocating for specificity and/or selectivity of these drug responses. Our findings suggest that by characterising the transcriptomics of the tissue cores we can identify molecular signatures which can potentially be predicting drug response.

### Sample strata may be predicted using a transcriptomics-based ML model

**Fig. 5:**
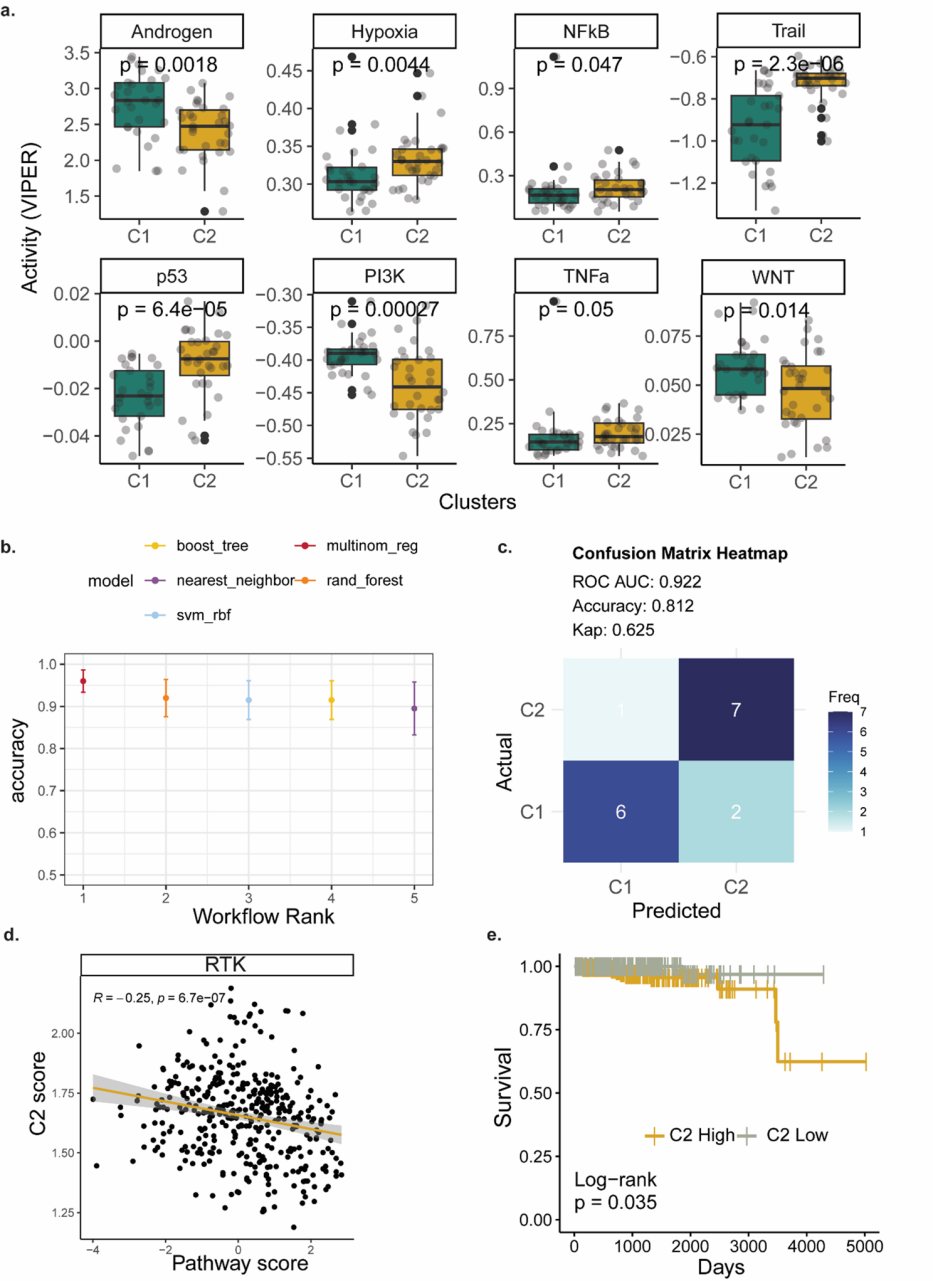
Patient samples can be stratified using a transcriptomics-based ML model and the clusters defined in an unsupervised manner (**a**) Pathway activity comparison reveals significantly differentially active signaling between the two clusters (Wilcoxon test). (**b**) Cross-validation ranking of the different workflows highlights multinomial regression as the methods with the highest accuracy. (**c**) Confusion matrix of the predicted clusters versus the actual clusters in 25% of the sample cohort which was used as a test set. (**d**) C2 signature (top 50 DEGs) score versus RTK pathway activity shows a negative correlation in TCGA data (Pearson correlation). (**e**) C2 signature is associated with worse survival in TCGA data (log-rank test)

We then sought to explore if we can model the drug response utilizing the transcriptomics data and the identified clusters. We first estimated the pathway activity of all the samples using a set of well-defined signaling pathways and a robust computational approach. Comparing the pathway activity among the two clusters showed that 8 (out of 15 PROGENy pathways tested) were differentially activated between the two clusters (Fig. 5a). Notably, we observed Androgen, PI3K and WNT pathways more activated in C1 which showed higher resistance to crizotinib and ponatinib, suggesting a potential connection between the activation of these pathways and the drug responses. The pathway activities were then used as features to model the two identified clusters and subsequent drug response. To reduce the dimensionality of the data and model the drug response in a human-interpretable manner we opted to use pathway activity scores instead of gene expression. We tested multiple machine learning algorithms in the 75% of the cohort (*n* = 45) by cross-validation and identified multinomial regression as the most accurate model (Fig. 5b). We then tested the optimal model to the remaining 25% test set (*n* = 16) and correctly classified the significant majority of the samples (Fig. 5c). We, therefore, propose a novel approach combining clustering, pathway activity estimation and machine learning to stratify prostate cancer patients (Sup. Figure 10).

To further explore the translational value of the identified clusters and characterize the molecular drivers that differentiate them we performed differentially expression analysis among the cores of the two clusters. We identified 942 differentially expressed protein-coding genes (403 up- and 539 down-regulated in the C1 vs. C2 comparison, Sup. Figure 9c). We selected the top 50 differentially expressed genes of each cluster to estimate a signature score across the TCGA samples. The C1 signature is inversely correlated with EMT pathway (Sup. Figure 11a), while both signatures (C1 and C2) as associated with a series of molecular pathways, e.g. androgen and DNA damage (Sup. Figure 11b-c). Interestingly, C2 signature is inversely correlated with RTK signaling in the TCGA samples (Fig. 5d), indirectly validating the Crizotinib resistance the C2 samples show in our drug screen. Importantly, C2 signature is also associated with worse survival prognosis (Fig. 5e). Therefore, using the TCGA data we characterized C2 as a more aggressive subset of patients with resistance to Crizotinib, most probably driven by lower RTK signaling.

**Fig. 6:**
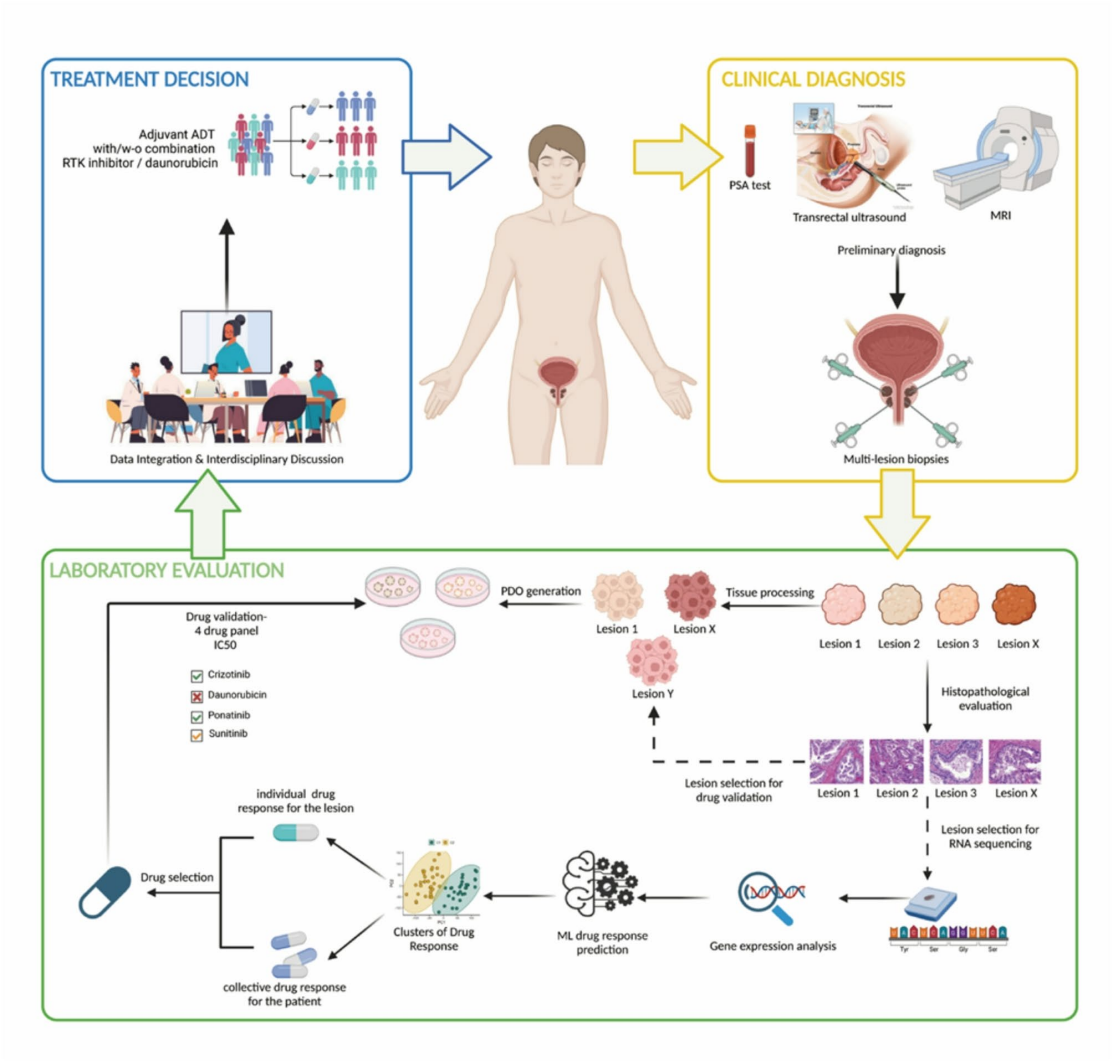
Multi-layer stratified oncology working model

Taken together, our findings suggest that primary tissue transcriptomics can be used to classify PCa patients into strata with distinct molecular features and response to drugs (Fig. 6). Importantly, the flexible nature of the proposed approach allows the revisiting of the clustering approach given more samples are available in the future, by re-estimating the optimal number of clusters and re-building the classification ML model.

A potential framework for patient-tailored strategies to guide clinical decision-making in prostate cancer. Upon obtaining positive results from diagnostic examinations such as PSA test, transrectal ultrasound, CT scan, and MRI, patients at risk for PCa receive a preliminary PCa diagnosis and will subsequently undergo prostate biopsy sampling. Twin biopsies from each lesion will undergo histopathological evaluation, and cryopreservation for later drug validation. The carcinoma lesions will be subjected to RNASeq utilising FPPE material, using our workflow. Gene expression profiles will be integrated into our established drug prediction ML model to predict PDO drug responses. The flexibility of the ML model also allows the revisiting of the clustering approach and classification based on addition of new samples. Both the collective drug response prediction across different lesions and the patient-specific drug response prediction will then be consolidated to determine potential drug candidates. Our ML model showed efficacy in predicting sensitivity to four compounds (crizotinib, ponatinib, daunorubicin and sunitinib). These drug candidates will then be validated in a dose dependent manner, using PDOs derived from the previously reserved biopsy samples. Subsequently, an interdisciplinary scientific board, consisting of specialists from diverse research and clinical backgrounds (such as oncologists, urologists, biologists, and bioinformaticians), will deliberate these findings to determine the optimal treatment strategy, such as one of the validated drugs in combination with adjuvant androgen deprivation therapy.

## Discussion

A lack of both experimental and patient-derived models for primary stage PCa represent a challenge in the field. We have previously shown that PDOs from primary PCa can be successfully established, while containing high tumor content and transcriptional similarity with matched tissues. However, the maintenance of the primary PCa PDO cultures is short-term, which is compatible with clinical decision making, yet not as a long-term experimental model. Models representing the multifocality and heterogeneity, commonly present in primary PCa, are particularly lacking in the field. Our study aims to address this by providing simultaneous characterization of histopathology, genomic, transcriptomic and functional drug responses, using matched mirror biopsies. In this study, we elucidated the molecular and functional heterogeneity of primary PCa and the potential mechanisms underlying drug response, utilizing PDOs. The innovative points that distinguish our study from prior PCa PDO work are (i) the use of spatially matched twin biopsies, (ii) integration of transcriptomics and drug response, and (iii) a predictive ML model based on FFPE tissue.

The developed transcriptomics-based machine learning drug prediction models, offers a preclinical approach with the potential to inform and enhance clinical trials aimed at targeted therapeutic investigations. Given that PDO generation is not routinely performed, the predictive model using routine histopathology and bulk RNASeq on FFPE, can be used to select compounds or stratify cases for targeted PDO testing.

Overall, our patient cohort is well representative of PCa heterogeneity, encompassing different clinical stages with multifocal lesions ranging from GS 6 to GS 10. Although there was a consistent rate of PDO formation and a predominant solid PDO morphology observed across tissues with varying histopathological features, the inherent histological and genetic heterogeneity of PCa were still retained in PDOs. This retention is underscored by the expression of both luminal and basal markers in PDOs, aligning with findings that suggest both cell types may be precursors to varying PCa stages [49–52]. Additionally, the shared genetic mutations between PDOs and their originating tissues underscore the promise of PDOs as a reliable model for depicting the complex genetic landscape of PCa. In many cases identification of somatic mutations was not achieved, even from histopathologically confirmed specific tumor areas, which could be attributed to low frequency of oncogenic mutations, or presence of mutations in regions not encompassed by our targeted DNA sequencing analysis.

The confounding effect between the absence of mutations and malignant phenotype at histopathology might be indicative of a transitional state between benign and tumor phenotypes, of undetermined yet features. Our findings are in line with the overall presence of low frequency mutations (below 3%) in the primary PCa [53] which could be identified only in larger cohorts, i.e. 26% of primary cases are not associated with a specific genomic subtype in the PRAD TCGA cohort [54]. The tumor heterogeneity within PCa necessitates a complex molecular classification of patients [55, 56]. While most drug-targetable mutations manifest at very early stages and can be readily detected through whole-genome sequencing [57] the slow progression of primary PCa -with only three recurrently mutated genes (ERG, PTEN, and SPOP) in over 10% of primary tumors [58]- coupled with our use of targeted genomic panel might be underlying reasons for the limited efficacy of genomic profiling in capturing the heterogeneity of our primary PCa cohort [59]. Therefore, there is an intrinsic limitation on our genomics analysis which could be mitigated in future studies by recruiting larger cohorts. Moreover, the presence of PCa being a type C tumor with more copy number alterations rather than single nucleotide variations advocates for the use of whole exome sequencing over a targeted panel.

Gene expression profiling of independent tumor foci can bridge the gap between genomics and phenotype and identifying molecular features associated with tumor or benign states. PDXOs and PDOs show high trancriptomic correlation with their originating tumor tissues as we have previously shown [17]. Thus, we hypothesized that RNA sequencing on the parental tissue in combination with PDO drug testing may represent a suitable approach to evaluate drug efficacy and to prioritize compounds for in vitro validation, but also to associate gene expression-drug associations and identify drug vulnerability mechanisms. From our previous study, a PDXO-based automated screen revealed efficacy of several TKI drug compounds (e.g. bosutinib, sunitinib, ponatinib) in primary and advanced PCa [17]. In the present study, we highlighted the consistent high efficacy of crizotinib, which stood out among 3 most effective TKIs (crizotinib, ponatinib, and sunitinib) observed in our cohort across heterogenous tumor lesions. Phosphoproteomics have revealed a higher tyrosine kinase phosphorylation activity in primary PCa versus benign tissues, as well as distinct phosphorylation signatures that are stage-specific ”footprints” for primary or CRPC stage [60, 61] which explains the efficacy of different TKIs in our PDO cohort targeting multiple Receptor Tyrosine Kinases. TKIs are increasingly being considered for and shown efficacy in primary PCa treatment [60, 62]. Activation of RTKs stimulated by growth factors, in specific cell populations has been implicated with promoting oncogenic signals and even selecting of androgen-independent cells [63, 64].

Several RTK receptors and their ligands were differentially expressed among our two Clusters C1 and C2, their upregulation has been found to be caused by hypoxia (e.g. VEGF-R and ligands) [65]with hypoxia pathway activity found significantly higher in C2 (sensitive to crizotinib and ponatinib). The high sensitivity of PCa PDOs to crizotinib could be due to abnormal cMET RTK activation, through ligand upregulation, MET mutations, gene amplification, and transcriptional upregulation [66]. In our data, cMET transcriptional upregulation, rather than genomic aberration, seems to be the cause of high efficacy of crizotinib [67]. High cMET-HGF signaling is also known to act through PI3K/AKT pathway [68] in our data HGF ligand levels were unchanged but MET transcript levels where higher in C1, in line with higher PI3K/AKT in C1 cluster.

As a target for crizotinib, cMET is not only confirmed to be overexpressed in primary PCa [69] specifically in malignant intermediate cells (CK5+/CK8++) [63] but also exhibits an inverse correlation with AR expression [70, 71]. Given that ADT remains the SOC for PCa, the removal of androgens in PCa patients could further augment cMET expression and driving cancer progression [63, 72]. This further supports the efficacy of crizotinib in PCa cell lines, organoids, and PDX models [17, 73, 74]. Our data indicate that crizotinib may represent a therapeutic option already at the primary, hormone sensitive stage of the disease in a setting of MET transcriptional upregulation.

Despite the general efficacy exhibited by the three TKIs in our cohort, the spatial molecular variations and varied drug sensitivities in primary PCa emphasize the importance of matching spatial sampling with drug response [12, 13]. Furthermore, there is a clear need to evaluate drug responses from multiple lesions comprehensively [75] as these insights will be instrumental in guiding treatment decision-making. Given the timeliness and capability of PDOs to capture the characteristics of parental tumors, we have, in conjunction with drug response data from PDOs and transcriptomic profiles from parental tissue, established two transcriptomics-based ML models. During the development of our models, we explored various combinations including histopathological features, genomic profiles, and clinical parameters. However, only transcriptomics consistently emerged as the most effective contributor to predictions [76, 77]. This might be attributed to the capacity of transcriptomic features to more comprehensively characterize intrinsic tumor signaling patterns, enabling the ML model to better capture drug target information [78, 79]. The features that were most important for the prediction where commonly aberrated pathways in PCa such as androgen, p53, hypoxia, PI3K which were significantly differentially active between the two identified clusters. Wnt pathway modulation is also found along with differential expression of Wnt related RTKs, as well NFkB, Trail and TNFa inflammation and immunomodulatory pathways. These clear differences in pathway activity motivated our decision to model the two strata using pathways instead of gene expression. Pathway activity allowed the biology-driven reduction of the dimensionality of the data and accurate modelling of the identified clusters. In ML applications there is always a risk of overfitting, especially given the relatively limited number of patient cohorts. We mitigated this risk with our pathway activity-based approach and by employing the splitting of the dataset into two sets (training and unseen test set) of adequate size (*n* = 45 and 16, respectively).

While the research study has deepened our understanding of the heterogeneity in multifocal PCa, particularly its functional variability, and has partially elucidated the potential drug response mechanisms for repurposing FDA-approved compounds for use in PCa, our study is not without limitations. Beyond the limitations in capturing heterogeneity through genomic profiling, the degraded quality of DNA and RNA due to FFPE processing further complicates molecular characterization. Besides, our method of macroscopically sampling distinct cores might have limited the precision in collecting tumor foci. Techniques such as MRI-guided biopsies could potentially improve the accuracy of tumor sampling in future studies. Moreover, a significant constraint in our study was the limited amount of available material. This limitation restricted our ability to thoroughly investigate intra-patient reproducibility, test multiple drug concentrations, and explore drug response mechanisms in more depth. Despite this, the drugs and concentrations selected, were based on dosage requirements due to 3D culturing [80] and our prior medium-throughput organoid drug screens [17] and were effective in distinguishing high and low responders within our cohort. While PDOs have shown promise for preclinical drug validation, most of the drugs we tested have not been FDA-approved for PCa treatment. As such, our drug response and prediction findings cannot be directly applied to patients at present. However, this limitation may soon be alleviated with ongoing Phase I clinical trials for crizotinib in advanced PCa (*ClinicalTrials.gov* #NCT02207504), in combination with enzalutamide (NCT02465060), for ponatinib and other drugs included in our panel for CRPC treatment (erlotinib, doxorubicin, everolimus, #NCT03878524), as well as the promising results sunitinib has already achieved in clinical trials (#NCT00631527 and # NCT00672594). Based on our observations we envision that imaging-guided sampling together with neoadjuvant treatment with TKI inhibitors and ADT is a promising treatment approach that could circumvent radical prostatectomy for specific patient groups selected according to response biomarkers.

In summary, this study provides a detailed exploration of the complex molecular and functional aspects of multifocal primary PCa. It illuminates the correlation between gene expression profiles and drug response, offering potential biomarkers and therapeutic targets for future investigations. Moreover, it introduces ML models that allow integration of new samples and reclassification of subtypes of responses, which can potentially assist in clinical decision-making, advancing the evolving landscape of PCa treatment towards precision medicine.

## Supplementary Information

Below is the link to the electronic supplementary material.

Supplementary Material 1

Supplementary Material 2

Supplementary Material 3

## Data Availability

All unique materials are readily available upon request to the corresponding authors. The RNA Sequencing data generated in this study are available at the Gene Expression Omnibus (GEO) under accession number GSE294532. The code to reproduce the analyses and the figures can be found at https://github.com/pchouvardas/Kang_Chouvardas_2025.
