# Supplementary material for: Multi-layer stratified oncology platform utilizing transcriptomics, prostate cancer organoids, and modeling of drug response
Source: J Exp Clin Cancer Res. 2025 Oct 16;44:290. doi: 10.1186/s13046-025-03540-2 (PMC12529805; doi:10.1186/s13046-025-03540-2)
Supplement: Supplementary file 1 — Supplementary Material 1 [file 13046_2025_3540_MOESM1_ESM.pdf]

Supplementary Figures & Legends

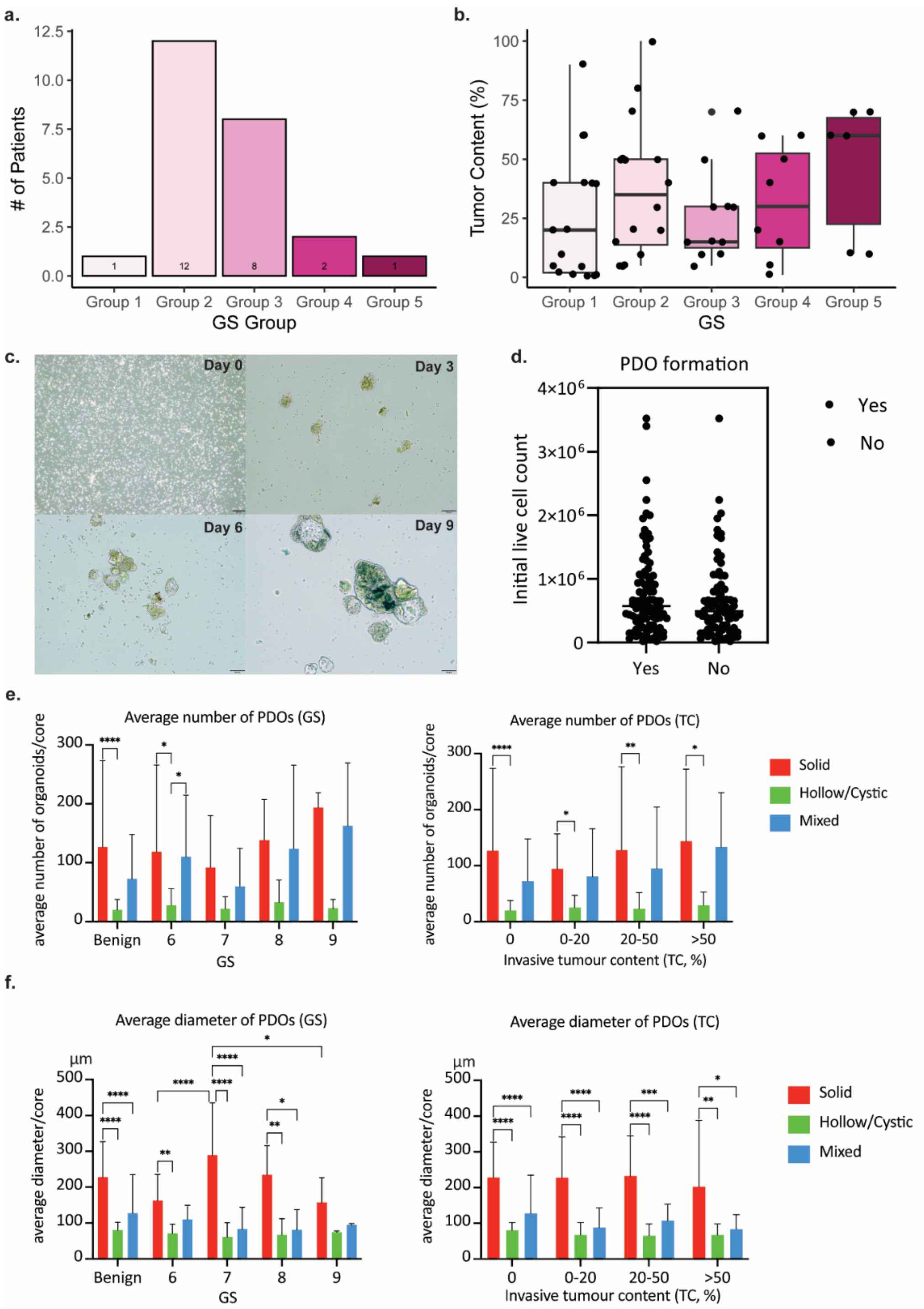

Sup. Fig. 1. PDO quantification and forming efficiency, related to figure 1.

(a) Overall pathological Gleason grade group reported per patient (left) based on pathologist`s diagnosis after radical prostatectomy.

(b) Distribution of Gleason grade group in relation with tumor content percentage (TC%), in the evaluated tumor cores.

(c) Representative brightfield images of the kinetic of initial organoid formation from tissue dissociation (Day 0) and single cell suspension to progressively organoid structure and increase in size (Day 3, 6 and 9). Scale bars 50µm.

(d) PDO formation versus no formation in relation to initial live cell count obtained from each tissue core directly after dissociation (unpaired t-test, p-value=0.2658).

*(e) Quantification of number of deriving PDOs, of distinct PDO morphologies (solid, hollow, mixed) in histopathologically defined benign or tumor cores, with varying GS and TC% (*p-value<0.5, **p-value<0.1, ***p-value<0.01, ****p-value<0.001).*

*(f) Quantification of diameter of distinct PDO morphologies (solid, hollow, mixed) in histopathologically defined benign or tumor cores, with varying GS and TC% (*p-value<0.5, **p-value<0.1, ***p-value<0.01, ****p-value<0.001).*

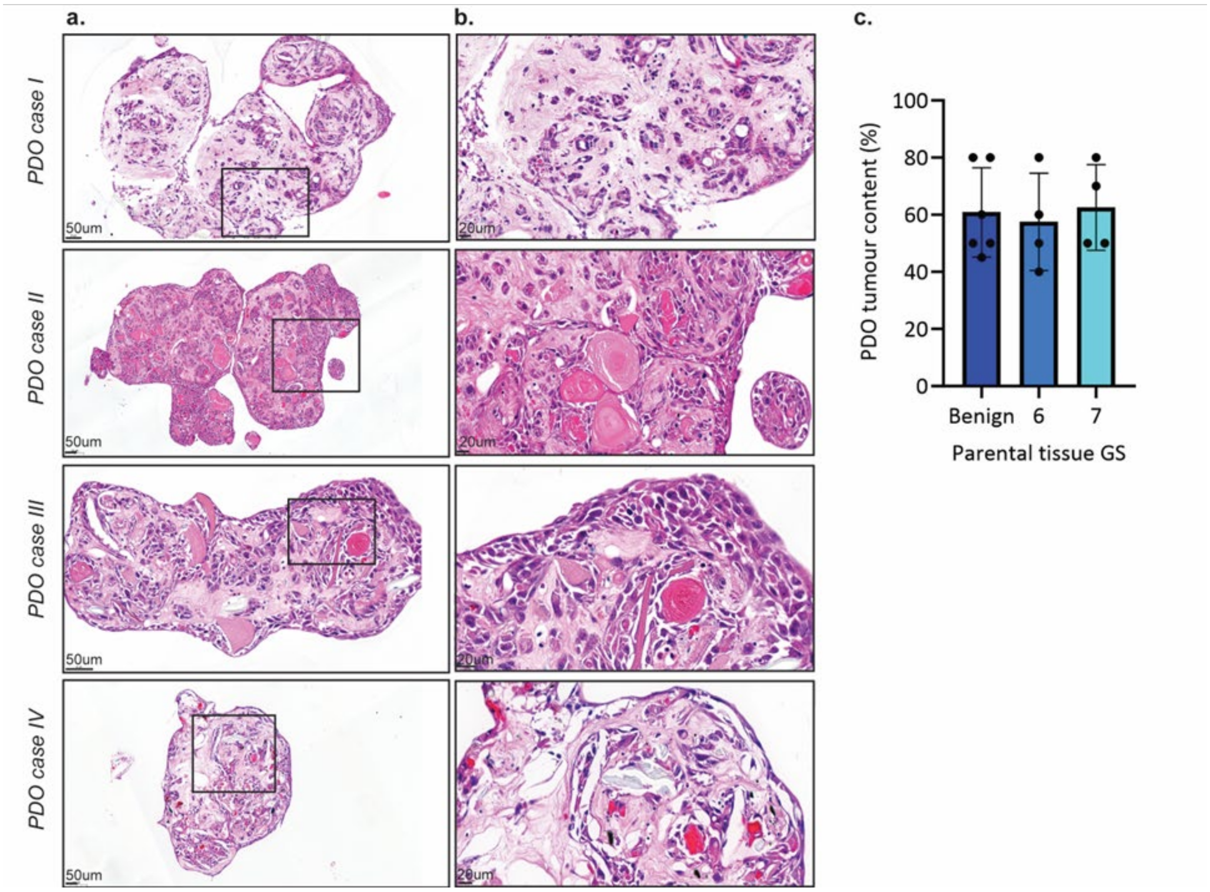

Sup. Fig. 2. Histopathology of PDOs, related to Fig.1.

(a) Tumor organoid histology of hematoxylin-and-eosin (H&E)-stained FFPE samples (cases I-IV). Scale bar: 50 µm. Rectangular boxes indicate areas with tumor features, shown at higher magnification in (B).

(b) Higher magnification images. The H&E stain allowed us to distinguish these different aspects, therefore complementary stains were not necessary for this description. Specifically, the outer membrane was mostly made of epithelial cells, corresponding to tumor cells, given their atypia. The nucleus –plasma relation is increased, with many cells displaying hyperchromasia in the nucleus and the nucleus membrane was often irregular. The stroma is acellular but edematous with very fine and rare structures resembling pseudoglandular structures. Scale bar: 20 µm.

(c) Comparison of PDO tumor content with GS of the tissue of origin. Median tumor content quantified from n=3-12 organoid structures per case, from n=6 benign tissue cores, n=8 tumor tissues, n=4 GS6, n=4 GS7.

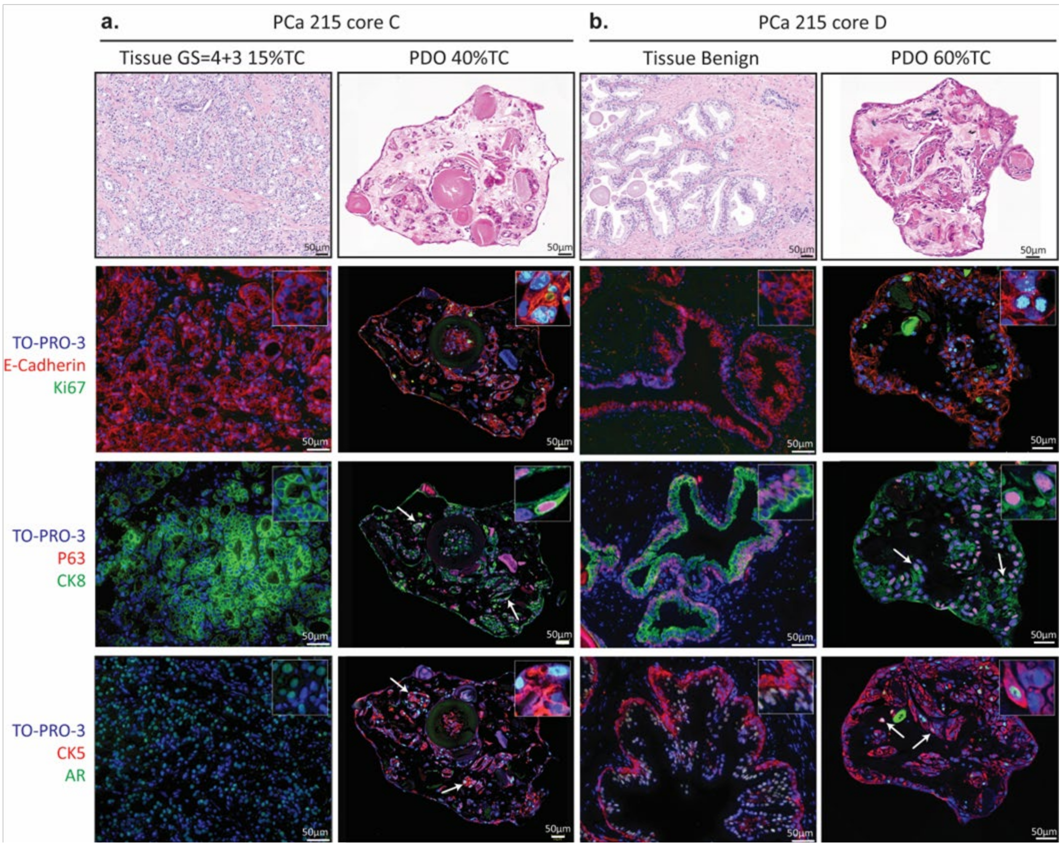

Sup. Fig. 3. Prostate cancer (PCa) patient-derived organoids (PDOs) recapitulate multifocal primary PCa histopathological features in vitro, related to Figure 2

Representative core tissues and matching PDOs from the same case.

(a) Hematoxylin-and-eosin (H&E) and immunofluorescent staining of PDOs and parental tissue from a histopathologically defined tumor core (core C). Top panel: proliferation marker Ki67 (green), epithelial marker E-cadherin (red). Middle panel: luminal marker cytokeratin-8, CK8 (green), basal marker p63 (red). Bottom panel: luminal marker androgen receptor AR (green),

basal marker cytokeratin-5, CK5 (red). TOPRO-3 (blue) marks the nuclei. White arrows indicate examples of cells expressing both basal and luminal markers. Scale bar: 50 µm.

(b) H&E and immunofluorescent staining of PDOs and parental tissue from a histopathologically defined benign core (PCa215 Core D).

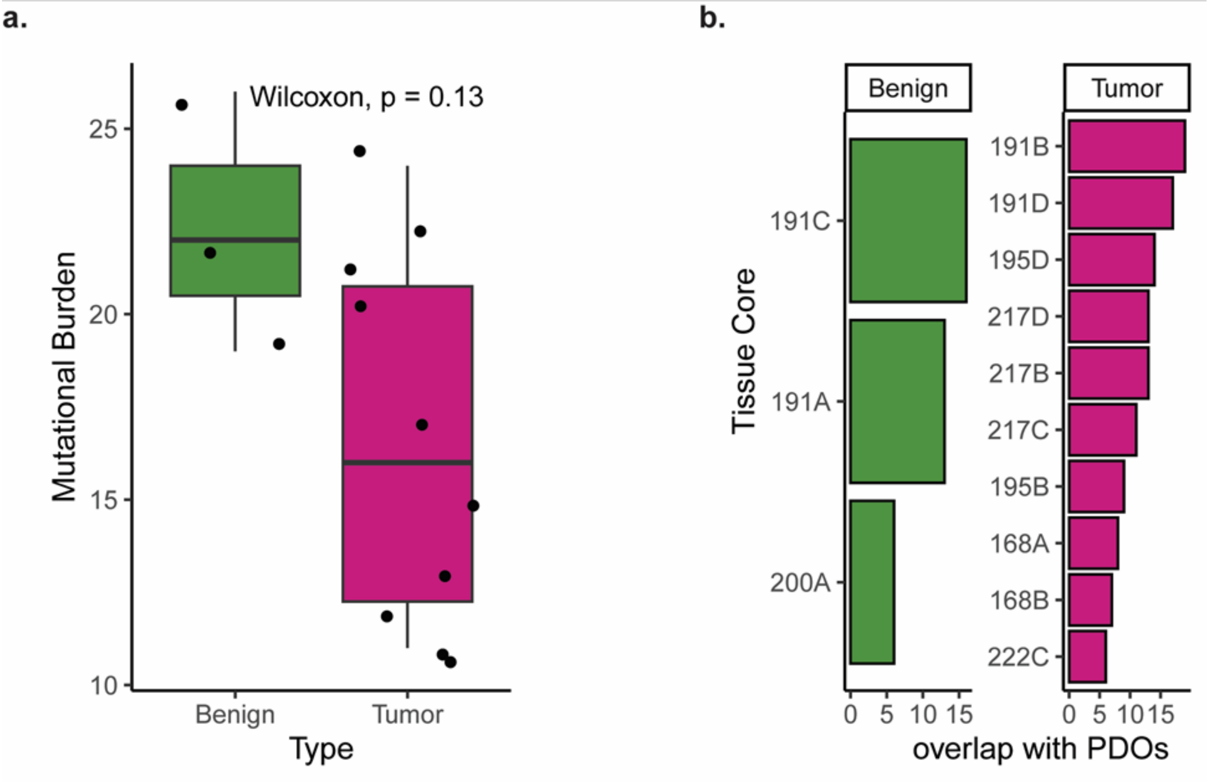

Sup. Fig. 4. Mutational characterization of multifocal PCa tissue cores and organoids without matched blood control.

(a) Distribution of mutational burden on tumor and benign cores as defined by pathological evaluation (Wilcoxon test, p = 0.13).

(b) Overlap of somatic mutations among matched cores and organoids (N=13) of different patients.

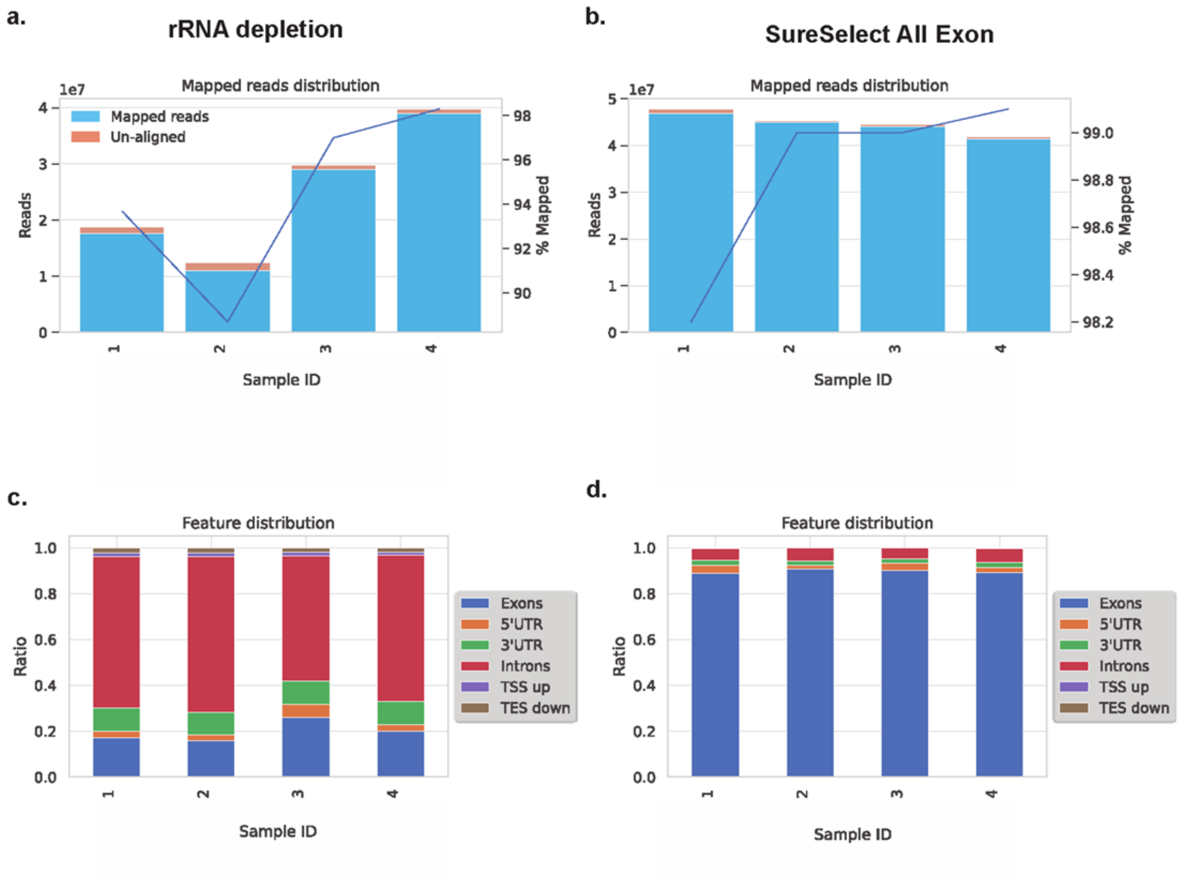

Sup. Fig. 5. Comparison of library preparation methods for FFPE Transcriptomics

(a) Mapped and unaligned read distribution after rRNA depletion.

(b) Mapped and unaligned read distribution after SureSelect All Exon.

(c) Feature distribution across different transcript regions after rRNA depletion.

(d) Feature distribution across different transcript regions after SureSelect All Exon. UTR: untranslated region; TSS: Transcription Start Site; TES: Transcription End Site.

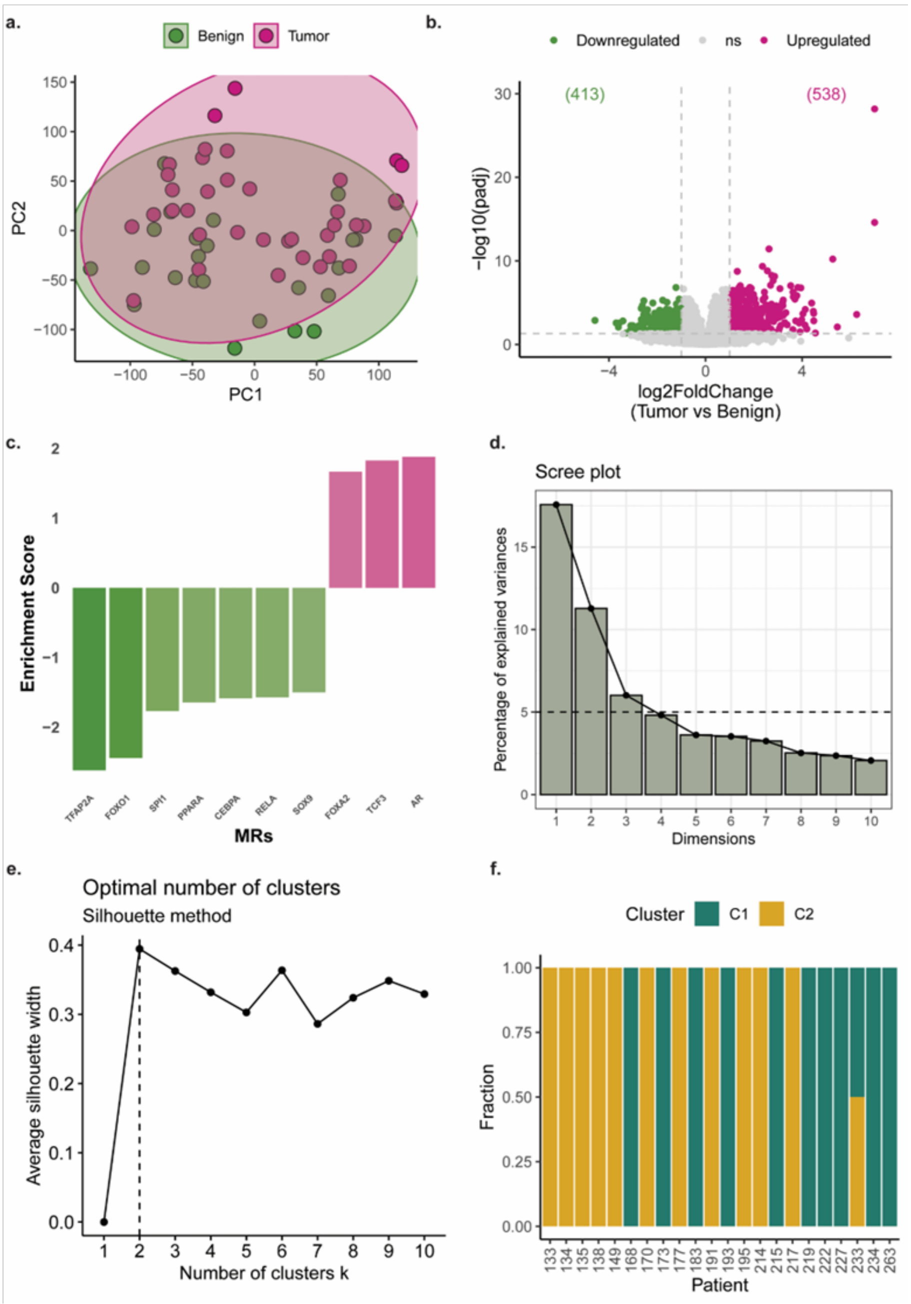

Sup. Fig. 6. Transcriptomic signatures on native PCa tissues cluster independently from benign/tumor origin, and uniform intrapatient heterogeneity of multifocal lesions and benign areas.

(a) Principal component analysis (PCA) for gene expression in FFPE parental tissues colored by pathology evaluation.

(b) Volcano plot showing the differential expression results among tumor and benign cores.

(c) Master regulator (MR) analysis reveals PCa related MRs activated in the tumor cores.

(d) Clustering distribution among the cores of the same patients highlight that the unsupervised approach overcomes the intra-patient heterogeneity.

(e) Volcano plot showing the differential expression results among cluster 1 and cluster 2 cores.

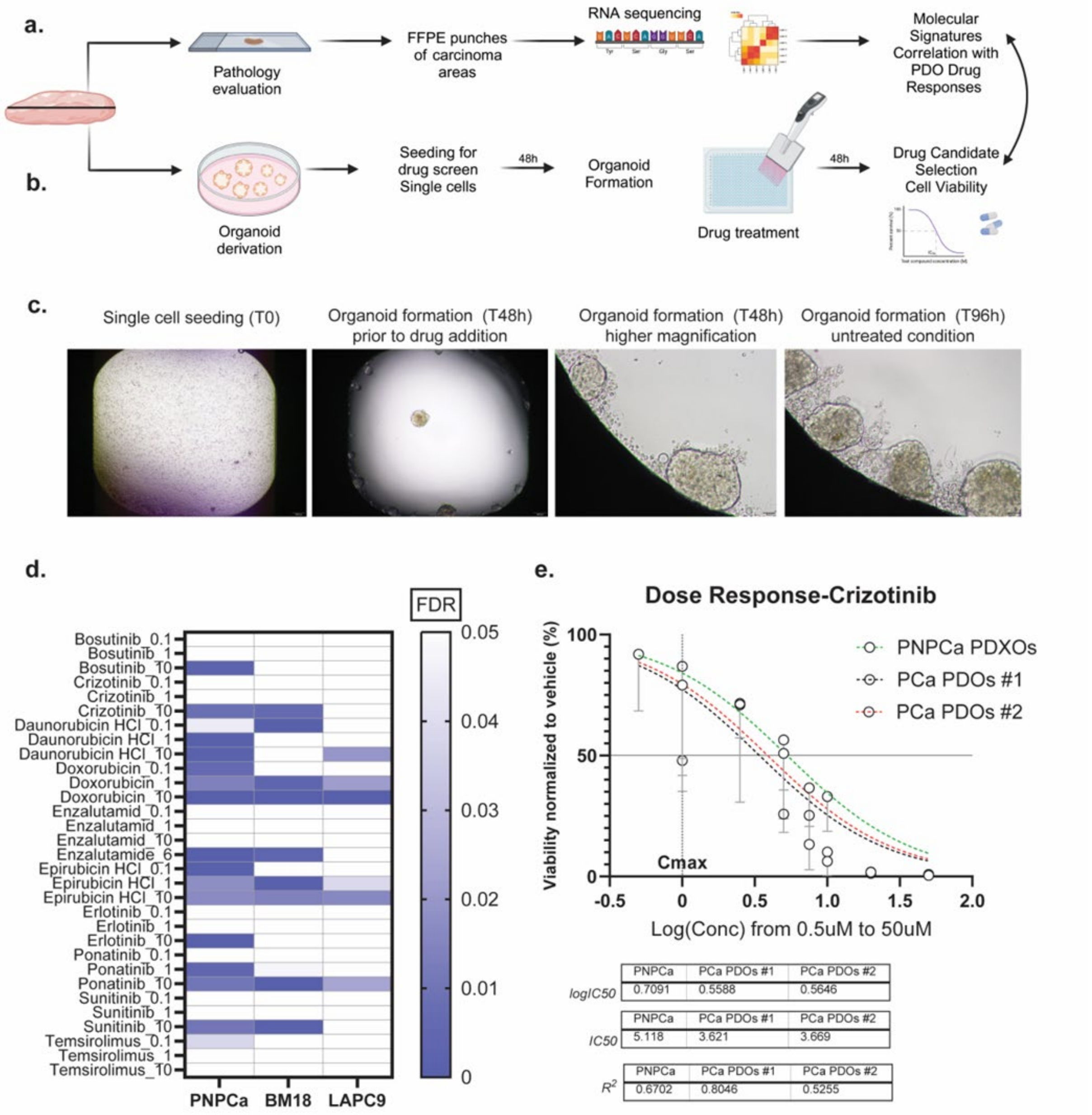

Sup. Fig. 7 Pipeline for correlation of molecular characterization of parental tissue with with PDO drug response on mirror biopsy

(a) Molecular signatures are obtained from carcinoma areas on FFPE tissues after pathologist`s evaluation and RNA Sequencing.

(b) The mirror biopsy of the FFPE block is used for patient-derived organoid generation

(c) Representative brightfield images of organoids during the different phases of the drug screen (from left to right; single cell seeding (T0) in 384-well ULA plates, organoids after 48

hours from seeding and just prior to drug treatment (T48) at two different zoom aspects and organoids after additional 48 hours (T96h) in control, untreated condition. Scale bar 50µm.

(d) Dose response of PCa organoids from Patient Derived Xenografts; PNPCa, BM18 and LAPC9 testing the selected drug panel at 0.1 uM, 1 uM and 10uM. Statistically significant effects are represented by FDR <0.05. The minimum concentration that was effective in 2 or more organoid models was selected for use in the present study.

(e) Dose response curve for crizotinib indicates that Cmax concentration (0.9-1uM) is ineffective for androgen sensitive PNPCa PDXOs as well as primary PCa PDOs. Consistently effective concentration (75% reduction in viability) for all models is between 7.5uM and 10uM. Organoid viability is normalized to the vehicle DMSO control of each sample. Logarithmic IC50, IC50 (uM) and R squared values are indicated.

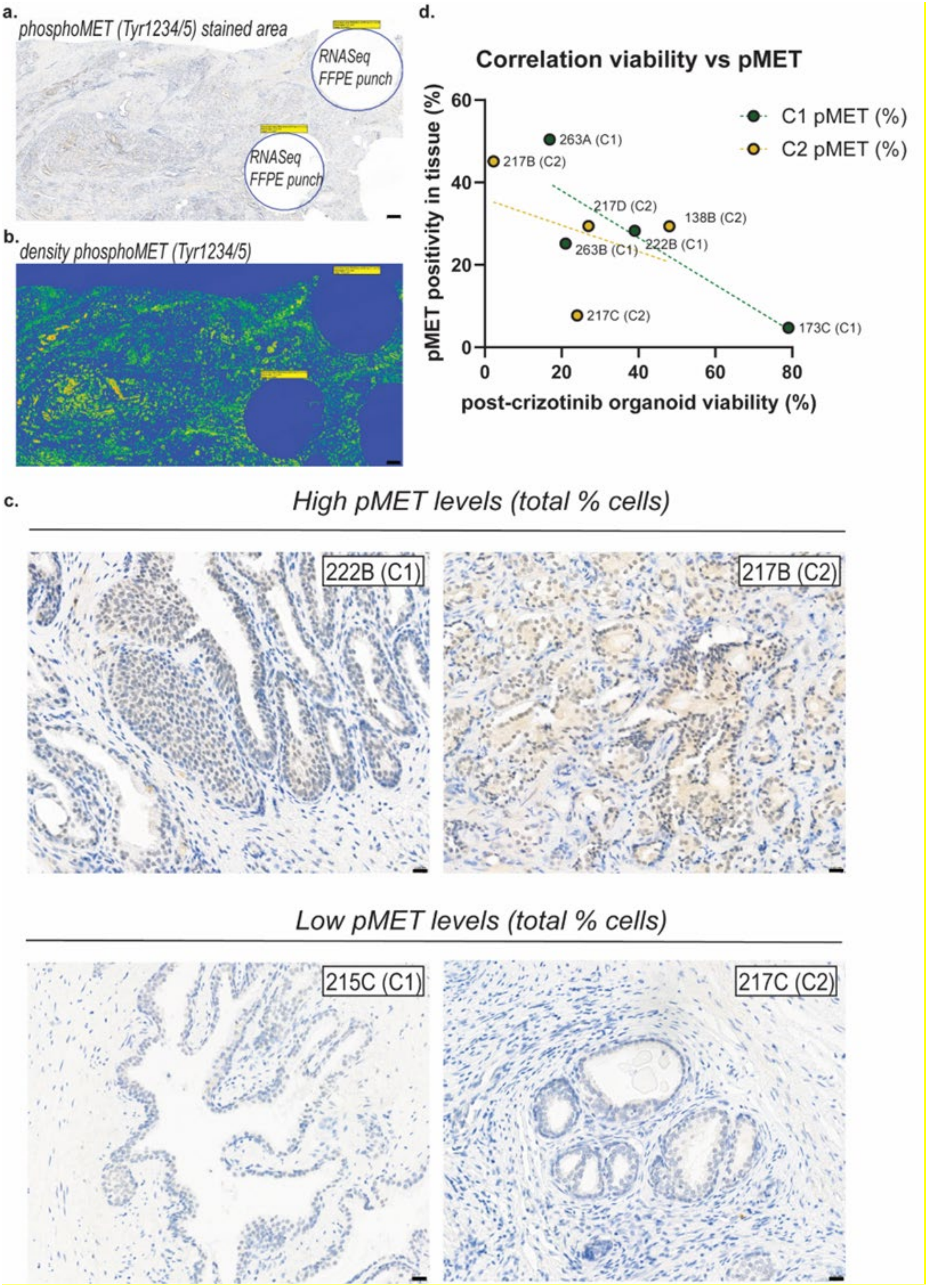

Sup. Fig. 8. Phosphorylated MET protein levels in tissues correlates with crizotinib response in matched PDOs

(a) Immunohistochemistry (IHC) for MET protein at phosphorylation of tyrosine residue Tyr1234 on primary PCa tissue cores. The same tissue sections were used for the initial RNA extraction used for the RNASeq and cluster profiling of the tissues. Scale bar 200 µm.

(b) Density of IHC pMET signal. Scale bar 200 µm.

(c) Representative IHC images of cases with higher (top) or lower pMET positive cells (%) (bottom). The sample code and its C1 or C2 cluster classification is indicated on each image. Scale bar 20 µm. Sample PCa222B is benign, samples 217B, 215C and 217C are from tumor areas.

(d) Scatterplot representing the organoid viability after crizotinib treatment and the matched pMET percentage of positive cells in the matched tissue (mirror biopsy). The organoid viability is shown as normalized vs its vehicle control (0-100%). The overall correlation is negative with r= -0.66 and p value= 0.071, while the individual cluster correlation for C1 is r= -0.8642 and p value= 0.136, and for C2 is r=-0.3871 and p value= 0.613.

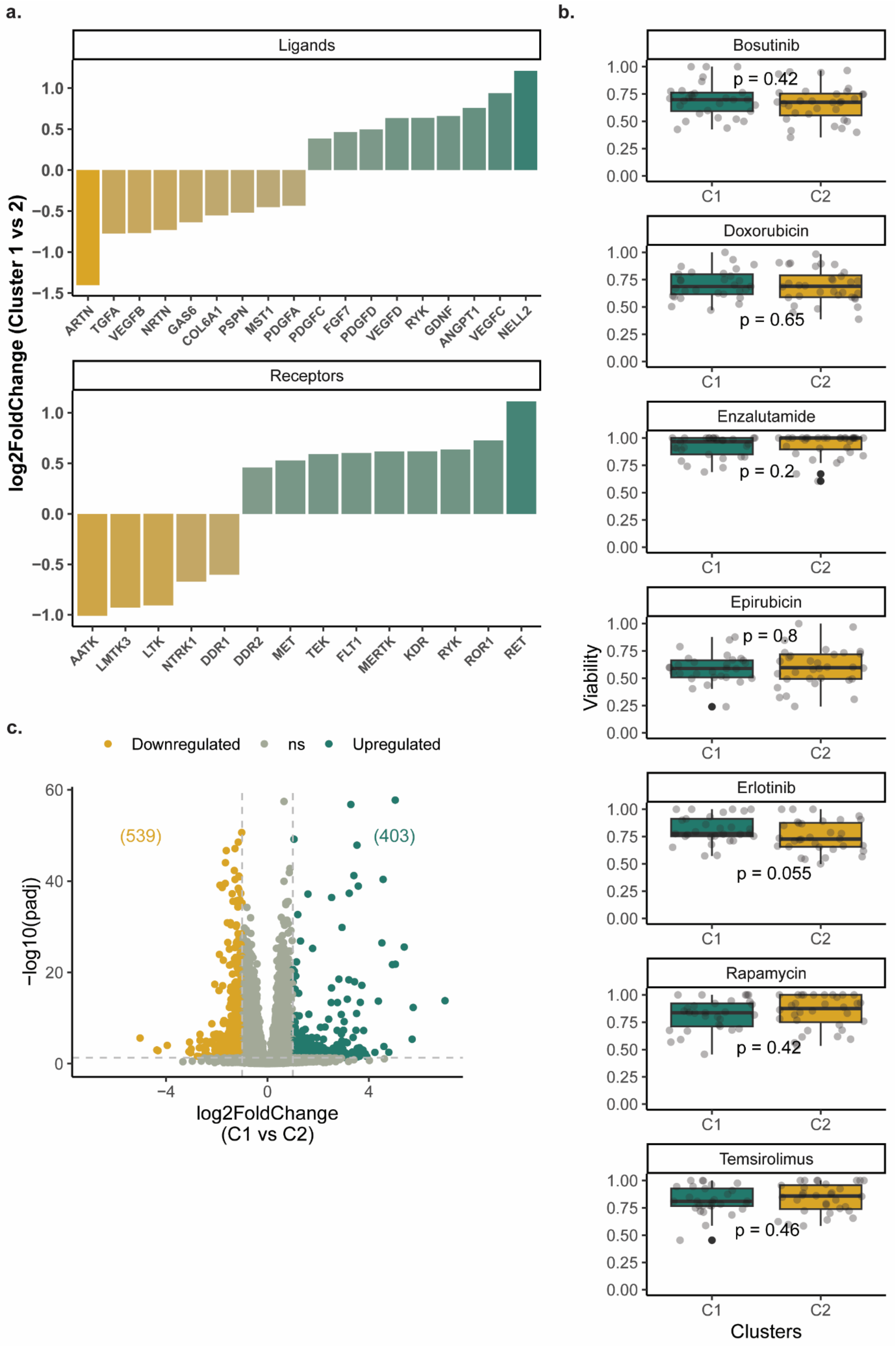
a.
Ligands
log2FoldChange (Cluster 1 vs 2)
ARTN TGFA VEGFB NRTN GAS6 COL6A1 PSPN MST1 PDGFA PDGFC FGF7 PDGFD VEGFD RYK GDNF ANGPT1 VEGFC NELL2
Receptors
AATK LMTK3 LTK NTRK1 DDR1 DDR2 MET TEK FLT1 MERTK KDR RYK ROR1 RET
b.
Bosutinib
p = 0.42
Doxorubicin
p = 0.65
Enzalutamide
p = 0.2
Epirubicin
p = 0.8
Erlotinib
p = 0.055
Rapamycin
p = 0.42
Temsirolimus
p = 0.46
Viability
Clusters
C1
C2
c.
Downregulated
ns
Upregulated
(539)
(403)
−log10(padj)
log2FoldChange
(C1 vs C2)

Sup. Fig. 9. PDO drug response with no difference between the two clusters, differential expression and characterization of RTKs and their ligands.

(a) Cluster 1 vs Cluster 2 differential expression results of RTK ligands (top) and receptors (bottom) receptors; all designated genes are statistically significant (adjusted p value ≤ 0.05).

(b) Drug compounds for which no differential organoid response was found, in comparison to tissue core stratification in clusters 1 and 2. Wilcoxon test C1 vs C2 was performed.

(c) Volcano plot showing the differential expression results among C1 and C2 cores.

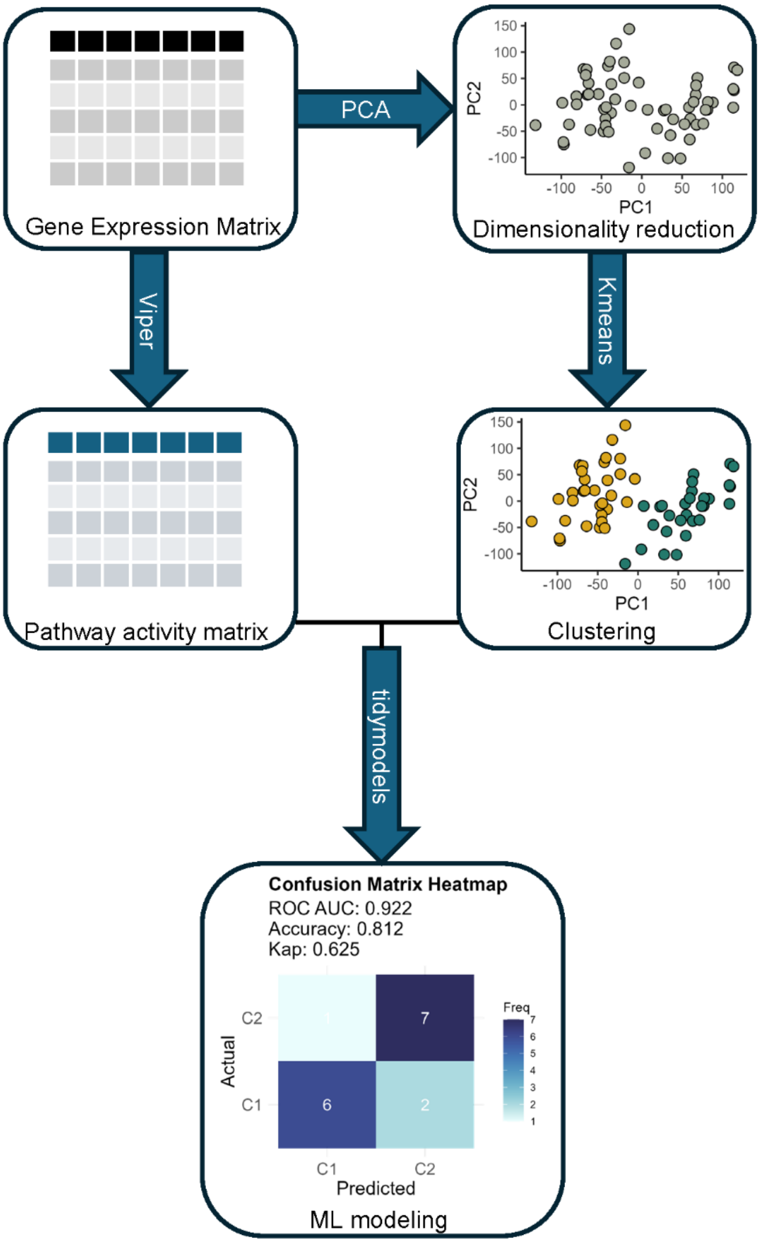

Sup. Fig. 10. Stratified oncology framework schematic showing the steps of clustering and machine learning based on gene expression and pathway activity, respectively.

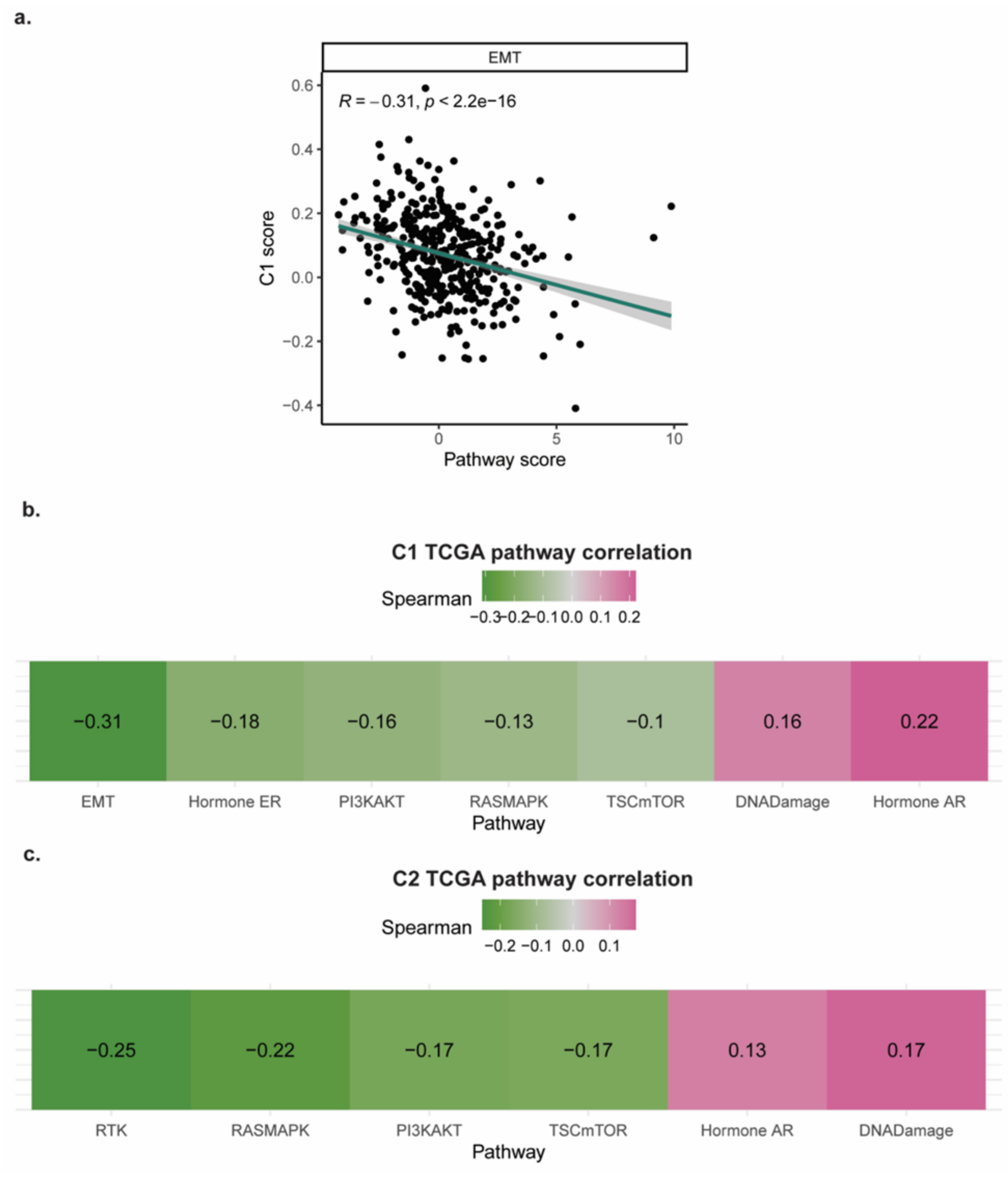

Sup. Fig. 11. Exploration of C1/C2 signatures in large scale cohorts.

(a) C1 signature is inversely correlated with EMT signature score in the TCGA samples (spearman correlation).

(b) Pathways which are significantly (fdr < 0.05) associated with C1 signature at the TCGA samples (spearman correlation).

(c) Pathways which are significantly (fdr < 0.05) associated with C2 signature at the TCGA samples (spearman correlation).
